# Constitutively elevated levels of SOCS1 suppress innate responses in DF-1 immortalised chicken fibroblast cells

**DOI:** 10.1038/s41598-017-17730-2

**Published:** 2017-12-13

**Authors:** E. S. Giotis, C. S. Ross, R. C. Robey, A. Nohturfft, S. Goodbourn, M. A. Skinner

**Affiliations:** 1 Section of Virology, School of Medicine, St Mary’s Campus, Imperial College London, London, W2 1PG UK; 2 Institute for Infection and Immunity, St George’s, University of London, London, SW17 0RE UK

## Abstract

The spontaneously immortalised DF-1 cell line is rapidly replacing its progenitor primary chicken embryo fibroblasts (CEFs) for studies on avian viruses such as avian influenza but no comprehensive study has as yet been reported comparing their innate immunity phenotypes. We conducted microarray analyses of DF-1 and CEFs, under both normal and stimulated conditions using chicken interferon-α (chIFN-α) and the attenuated infectious bursal disease virus vaccine strain PBG98. We found that DF-1 have an attenuated innate response compared to CEFs. Basal expression levels of *Suppressor of Cytokine Signalling 1* (chSOCS1), a negative regulator of cytokine signalling in mammals, are 16-fold higher in DF-1 than in CEFs. The chSOCS1 “SOCS box” domain (which in mammals, interacts with an E3 ubiquitin ligase complex) is not essential for the inhibition of cytokine-induced JAK/STAT signalling activation in DF-1. Overexpression of SOCS1 in chIFN-α-stimulated DF-1 led to a relative decrease in expression of interferon-stimulated genes (ISGs; MX1 and IFIT5) and increased viral yield in response to PBG98 infection. Conversely, knockdown of SOCS1 enhanced induction of ISGs and reduced viral yield in chIFN-α-stimulated DF-1. Consequently, SOCS1 reduces induction of the IFN signalling pathway in chicken cells and can potentiate virus replication.

## Introduction

The increasing occurrence of zoonotic infections attributable to avian viruses such as avian influenza viruses H5N1 and H7N9, West Nile virus, Japanese encephalitis virus, eastern (and western) equine encephalitis viruses, as well as avian *Salmonella* and *Campylobacter* bacterial species, has highlighted the need for well-established avian experimental models of infection and immunity. Limitations in the usage of embryonated chicken eggs (or chick embryo fibroblasts - CEFs), due to costly, time-consuming production processes or supply problems, hinder scaled-up procedures such as vaccine manufacturing, while alternative avian or mammalian cell substrates have several drawbacks, particularly due to restricted host- and receptor-specificity^1–3^.

CEFs have largely replaced embryonated eggs for vaccine production and viral infection studies as they are safe, proliferate well, are surprisingly consistent in terms of their expression profiles^4^ and provide high pathogen yield, albeit with increased cost, laborious manufacturing process and limited life span^1,3^. The requirement for avian cell lines in diagnosis and research, as well as for vaccine production, has shifted the focus of the scientific community towards deriving continuous cell lines that could eliminate recurring costs associated with CEFs. Avian cells are difficult to immortalise and new cell lines have been primarily developed using tumorigenic viruses, transforming oncogenes, or oncogenic chemicals, rendering them less suitable for vaccine production^2,5^. Embryonic stem cell lines such as duck EB66 and chicken EB14 are being evaluated for use in the vaccine industry, with the advantages that they are relatively genetically stable, have unlimited life span and circumvent disadvantages associated with tumorigenic cell lines^6,7^. Despite the availability of these new cell lines, large animal and human vaccine processes still rely heavily on CEFs as a first choice or as a certified alternative substrate for the propagation of many commercially available clinical vaccines such as those for measles and mumps (for example, MMR II, Merck), tick borne encephalitis (FSME IMMUN, Baxter) and rabies (RabAvert, Novartis)^3,8^.

An alternative to CEFs is the chicken fibroblast cell line UMNSAH/DF-1 (DF-1), which is gradually becoming a standard avian cell substrate. Derived originally from 10-day-old East Lansing Line 0 (ELL-0) eggs^9^, DF-1 is possibly the only readily available, spontaneously-immortalised, endogenous virus-free avian cell line that exhibits high transfection efficiency and a high proliferation rate while, at the same time, supporting satisfactory propagation of a broad range of avian viruses^10,11^. DF-1 cells have been extensively used for the propagation and/or study of various avian viruses, including avian influenza virus such as the highly pathogenic Eurasian H5N1 and H7N1 subtypes^12^, avian leukosis virus^10^, avian sarcoma leukosis virus (ASLV)^13^, fowlpox virus^14^, Marek’s disease virus^15^, infectious bursal disease virus (IBDV)^16^ and avian metapneumovirus^17^. Phenotypically, DF-1 cells are characterized by a suppression of cell death pathways (consistent with their immortal hyperproliferative phenotype^18^), dysfunctional cell proliferation-related genes p53 and E2F-1, as well as defective antioxidant gene expression^11,19,20^.

Compared with their progenitor CEFs, DF-1 have enhanced growth potential^18^, smaller morphology^21^ and can support comparable or even higher replication of IBDV, ASLV, avian influenza and some other viruses^12,13,16^. High viral replication in DF-1 implies that viruses (even attenuated vaccine strains) are not efficiently restricted by the cells’ antiviral innate immunity. This is despite reports that DF-1 readily express known interferon-stimulated genes (ISGs), potentially with antiviral activity, following stimulation with recombinant chIFN-α or, to lesser extent, with recombinant chIFN-β^22^. We hypothesised that the type I IFN-induction and/or signalling pathways in DF-1 might be dysregulated compared to CEF, compromising the innate response to viruses and thereby permitting their replication. However, although the constitutive gene expression profile of DF-1 relative to CEF has been compared^18^, their induced innate responses have not been compared directly.

Here we demonstrate, using microarrays, that DF-1 do indeed mount an operational type I IFN response following stimulation with recombinant chIFN-α or infection with a highly immunogenic attenuated vaccine strain of IBDV (PBG98) that was employed as a model pathogen to evaluate IFN induction and signalling. However, the relative number and expression levels of ISGs in DF-1 cells displayed an attenuated innate response compared with primary CEFs. Importantly, in DF-1 we observed that the regulatory ISG, SOCS1 (*Suppressor Of Cytokine Signaling*), was barely induced by IBDV infection or by IFN (by only 1.8 and 2.5 fold, respectively), which is to far lesser extents (23 and 8 fold less, respectively) than in CEFs. We attributed this to the high constitutive level of expression of SOCS1 we observed in DF-1. Kong *et al*.^18^, who published after conduct and analysis of our microarrays, also observed that constitutive levels of expression of SOCS1 in DF-1 were elevated relative to CEFs but they did not investigate IFN-stimulated gene expression and could therefore not appreciate the scale and significance of that observation.

We further demonstrate that the elevated constitutive expression of SOCS1 in DF-1 cells in turn attenuates IFN signalling and antiviral immunity. We also found that viral replication could be facilitated or impeded by transient up or down modulation, respectively, of SOCS1 in CEFs and DF-1 cells, respectively.

Although we have reservations concerning the comparison of the DF-1 cell line with the relatively mixed population of cells represented by CEFs, this study addresses the practical issue that in the past there have been (and continue to be) many studies of virus infection and innate responses in CEFs but that increasingly the same studies are more likely performed with DF-1. Our results will help researchers compare and understand those two types of study.

## Results

### Microarray analysis identified enhanced cell proliferation as well as repressed inflammation- and apoptosis-related gene expression in DF-1 cells

Comparison of untreated CEFs and DF-1 cells identified 856 transcripts that are more highly expressed in DF-1 cells than in CEFs, and 1747 transcripts that are more highly expressed in CEFs than in DF-1 cells (Supplementary Table 1). These data, obtained using the 35 K Affymetrix Chicken Genome Array, are consistent with those obtained using the 44 K 60-mer oligonucleotide Agilent chicken microarray, as reported by Kong *et al*.^18^ after we had already obtained and processed our results.

The resulting lists of differentially expressed transcripts were further analysed by the MetaCore^TM^ pathway enrichment analysis tool (Clarivate Analytics). The top 10 ranking canonical pathway maps associated with the upregulated transcripts in DF-1 cells are listed in Table 1. Upregulated transcripts were associated with critical processes that control progress through the eukaryotic cell cycle and the initiation of mitosis, such as spindle assembly and chromosome segregation that function at the G2-M transition (CDK1, cyclin A, importin-alpha, KNSL1), the anaphase-promoting complex (CDC20, Aurora-A, Nek2A CDK1, CKS1) activity, cell proliferation (BubR1) and DNA repair (BRCA1, BRCA2). Transcripts that were overexpressed in CEFs relative to DF-1 cells (Table 2) were associated with extracellular matrix (ECM) and cytoskeleton remodelling involved in embryonic development (MMP-9, MMP-13, TIMP3, PLAU, Keratin-5, −7, −14, −19), cell adhesion (E-cadherin, VE-cadherin) and signalling cascades (FGF, plasmin, TGF-β). In addition, many inflammation-associated transcripts (IL-6, IL-18, IL-1R1, COX2, TLR2, TGF-β2) and apoptosis regulators (caspase-3, lamin A/C, bCL2) were found to be repressed in DF-1 cells compared with CEFs.

**Table 1:** Top 10 statistically significant MetaCore pathway maps associated with upregulated genes in the comparison of unstimulated DF-1 versus CEF cells.

Pathways	*p*-value	Molecules
The metaphase checkpoint	2.831E-17	Nek2A, BUB1, Rod, MIS12, Aurora-A, PLK1, HEC, CDCA1, CDC20, CENP-C, CENP-F, Zwilch, ZW10, Survivin, CENP-E, BUBR1
Role of APC in cell cycle regulation	1.246E-13	Nek2A, BUB1, Tome-1, Geminin, Cyclin A, Aurora-A, PLK1, CDC20, Securin, ORC1L, CDK1 (p34), CKS1, BUBR1
Spindle assembly and chromosome separation	1.287E-10	Nek2A, Importin (karyopherin)-alpha, TPX2, Aurora-A, KNSL1, HEC, CDC20, Separase, ZW10, Securin, CDK1 (p34)
Chromosome condensation in prometaphase	4.671E-10	BRRN1, CAP-H/H2, CAP-G, Cyclin A, CAP-G/G2, Aurora-A, CAP-E, TOP2, CDK1 (p34)
Start of DNA replication in early S phase	1.895E-09	Cdt1, RPA3, Geminin, MCM3, E2F1, MCM10, ORC6L, ASK (Dbf4), ORC1L, MCM5
DNA damage_Role of Brca1 and Brca2 in DNA repair	1.887E-08	ATR, Rad51, MSH6, Bard1, Brca1/Bard1, Brca1, p53BP1, FANCL, Brca2
Transition and termination of DNA replication	1.835E-07	TOP2 alpha, Bard1, Cyclin A, Brca1/Bard1, Brca1, TOP2, FEN1, CDK1 (p34)
Nucleocytoplasmic transport of CDK/Cyclins	4.285E-07	Importin (karyopherin)-alpha, Cyclin A, CRM1, Cyclin D3, Cyclin D, CDK1 (p34)
Role of Nek in cell cycle regulation	7.845E-06	Nek2A, Tubulin beta, TPX2, NEK7, Aurora-A, HEC, CDK1 (p34)
DNA damage_ATM/ATR regulation of G1/S checkpoint	7.845E-06	ATR, BLM, Bard1, Cyclin A, Brca1, FANCL, Cyclin D

**Table 2:** Top 10 statistically significant MetaCore pathway maps associated with down regulated genes in the comparison of unstimulated DF-1 versus CEF cells.

Pathways	*p*-value	Molecules
ECM remodeling	7.669E-09	Matrilysin (MMP-7), Collagen II, MMP-13, TIMP3, Stromelysin-2, Collagen IV, SERPINE2, Nidogen, Osteonectin, EGFR, LAMA4, MMP-9, PLAU (UPA), Versican, Collagen III
Development_Regulation of epithelial-to-mesenchymal transition (EMT)	2.337E-08	IL-1RI, VE-cadherin, E-cadherin, TGF-beta 2, Caldesmon, Tropomyosin-1, PDGF-R-alpha, TGF-beta 3, Claudin-1, WNT, SLUG, EGFR, MMP-9, Frizzled, EDNRA, Bcl-2
Stimulation of TGF-beta signaling	1.363E-06	COX-2 (PTGS2), E-cadherin, TGF-beta 2, EGR1, TGF-beta, Tropomyosin-1, PI3K reg class IA (p85-alpha), TGF-beta 3, SLUG, Keratin 19, MMP-9, Tropomyosin-2
Cytoskeleton remodeling	3.722E-06	Keratin 5/14, PPL(periplakin), Keratin 7, Tubulin alpha, Trichoplein, Keratin 14, Plakophilin 2, Keratin 19, Keratin 5, Desmoplakin
FGF signalling	6.040E-06	E-cadherin, HBP17, PI3K reg class IA (p85), Glypican-1, PI3K cat class IA, FGF7, FGFR2, MMP-9, PLAU (UPA), FGF10, Alpha-catenin
Cell adhesion_Plasmin signalling	2.285E-05	TGF-beta 2, PI3K cat class IA, MMP-13, PI3K reg class IA, Collagen IV, LEKTI, TFPI-2, PLAU (UPA), Neuroserpin
Immune response_HMGB1/RAGE signaling pathway	2.569E-05	IL-6, PI3K reg class IA (p85), PI3K cat class IA, VCAM1, PI3K reg class IA (p85-alpha), IL1RN, Tissue factor, Secretogranin II, TLR2, MEF2C, MYOG
Development_TGF-beta-dependent induction of EMT via RhoA, PI3K and ILK.	3.925E-05	E-cadherin, TGF-beta 2, Caldesmon, PI3K reg class IA (p85), Tropomyosin-1, PI3K cat class IA, TGF-beta 3, Claudin-1, Actin, SLUG
Cell-matrix glycoconjugates	4.658E-05	CCL5, Fibulin-2, Fibulin-1, NCAM1, CRTL1, Tenascin-C, Elastin, MMP-9, Versican
Cytoskeleton remodeling_TGF, WNT and cytoskeletal remodeling	5.177E-05	p15, Matrilysin (MMP-7), PI3K cat class IA, Alpha-actinin, MMP-13, PI3K reg class IA, Collagen IV, p21, MELC, MYLK1, WNT, WIF1, Actin, PLAU (UPA), MLCK, Frizzled

**Figure 1:**
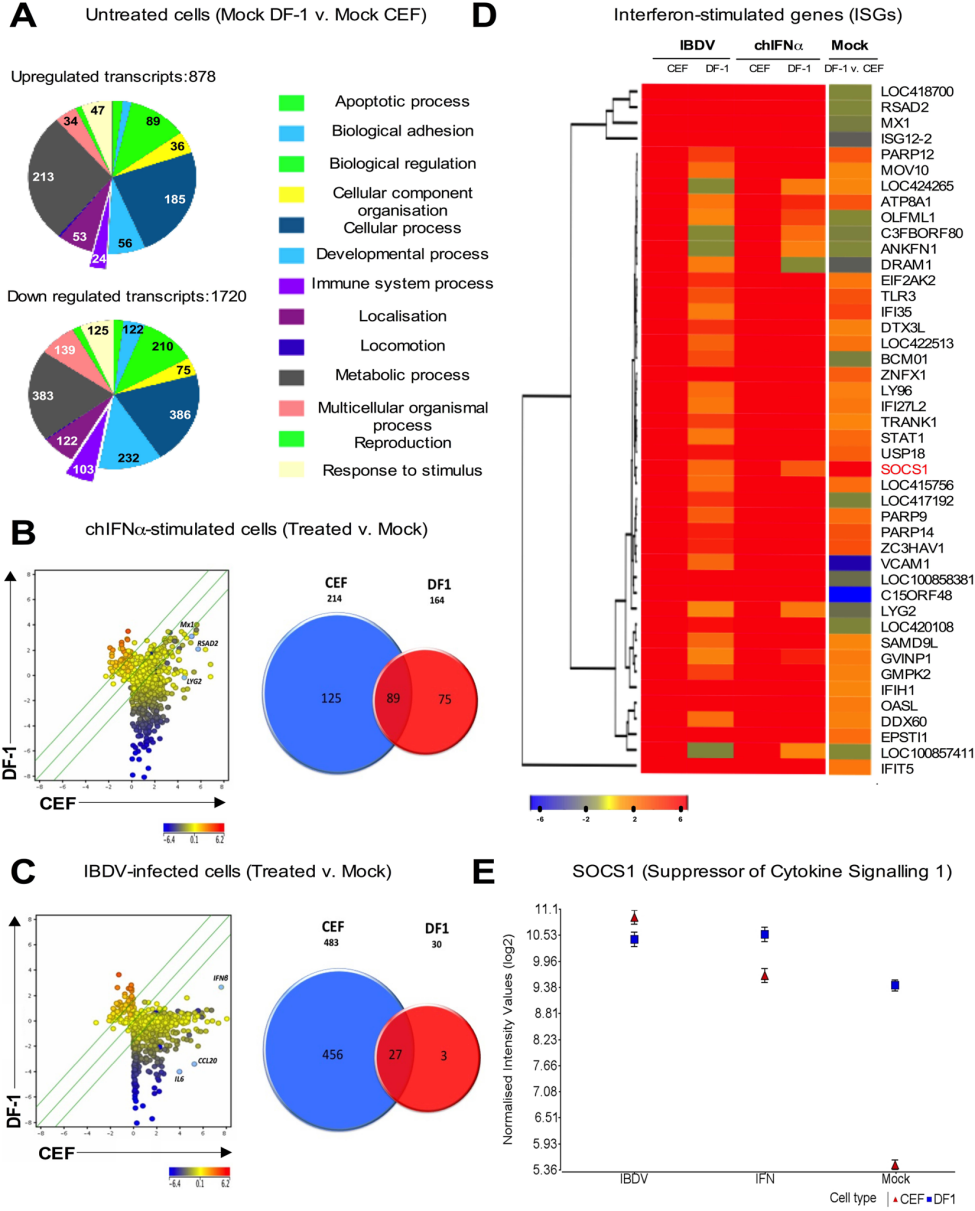
Microarray analysis shows that DF-1 have an attenuated innate response compared with CEFs. (**A**) Results from the microarray comparison between untreated DF-1 and CEFs. Pie charts represent the number of up- and down- regulated transcripts associated with different biological processes, assessed by Gene Ontology (GO) search and summarized according to their functions (PANTHER classification system). Scatter plots and Venn diagrams, showing extent of differential gene expression (Log2) and numbers of genes differentially regulated, respectively, in comparisons between CEFs and DF-1, either (**B**) treated with recombinant chIFN-α (1000 units/ml, 6 h), or (**C**) infected with IBDV (multiplicity of infection, MOI 5; 16 h). Scatter plots were generated using the Genespring scatter plot tool. Scatter plots show DF-1 (Y-axis) versus CEFs (X-axis) cells. The scale on the X- and Y-axis indicate expression levels (log2) and change in gene expression represented as a gradient of blue and red color for low- and high-expression intensity respectively. (**D**) Cluster analysis and heat-map showing differential expression of the top 45 ISGs (|fold change| ≥3.0 and FDR ≤0.01) as identified in CEFs following chIFN-α stimulation. ISGs were ranked by hierarchical clustering. Columns represent five comparisons, left to right: IBDV-infected CEFs and DF-1, chIFN-α-stimulated CEFs and DF-1 (each compared to their respective mock-treated control) and mock-treated DF-1 compared to mock-treated CEFs. Fold change in gene expression is represented by a blue (down-regulated) to red (up-regulated) colour intensity gradient. (**E**) Microarray expression data (log2 normalised intensity values ± Standard Deviation) for SOCS1 in, IBDV-infected, chIFN-α –treated or mock-treated CEFs or DF-1.

Functional categorization by Gene Ontology (GO) using the Panther classification database^23^ identified that the GO Biological Process most significantly overrepresented amongst upregulated (and the second most significant amongst downregulated) transcripts in untreated DF-1 relative to CEF was related to metabolic processes (with 24 and 22% of total transcripts upregulated and downregulated, respectively). Most of the transcripts were involved in energy metabolism and the regulation of metabolic processes in mitochondria. Innate and immune response-related transcripts constituted 3 and 6% of the total transcripts upregulated and downregulated, respectively, between the immortalized and primary cells (Fig. 1A).

Transcription factor analysis of array data for untreated DF-1 and CEFs, using the Metacore algorithm, identified a statistical overrepresentation (P < 0.001) of transcription factor binding sites for the transcription factor CREB1 (cAMP responsive element binding protein 1), which regulates diverse cellular responses including: proliferation, survival, differentiation and immune response. Other enriched motifs included c-*myc* (involved in cell growth and apoptosis), STAT-related transcription factors, NF-κB-related transcription factors (c-Rel, NF-AT1 & 2, p50, p52, and p65) and the cell proliferation-related E2F1 (E2F transcription factor 1), SP-1/3 (Sp1/3 transcription factors), and p53 (Supplementary Table 2).

### Compared with CEFs, DF-1 cells display an attenuated response to chIFN-α and IBDV infection

In order to characterize and compare the innate response of CEFs and DF-1, we profiled the cells in two ways: (i) by treating them with recombinant chIFN-α (1000 units ml^−1^) for 6 h, and (ii) by infecting cells with PBG98 (MOI 5, 16 h). Gene expression was analyzed by DNA microarrays and qRT-PCR. ChIFN-α initiated strong induction of ISG expression (213 transcripts) in CEFs while the response in DF-1 cells was attenuated, with induction of 164 ISGs (of which 89 were regulated in common with CEFs) (Fig. 1B). Levels of induced expression of ISGs (i.e. Mx1, RSAD2, LYG2) were consistently lower in DF-1 cells than in CEFs (Fig. 1B, Supplementary Table 1 and unpublished). IBDV PBG98 infection initiated robust regulation of host transcript expression in CEFs, with 345 transcripts upregulated and 138 transcripts downregulated. In comparison, DF-1 cells showed limited transcript induction (and no transcript repression) with 30 transcripts upregulated (i.e. chIFN-β, IL6, CCL20, Fig. 1C), 27 of which were regulated in common with CEFs. Comparison of the two cell lines shows different subsets of virus-induced transcripts and ISGs (Venn diagrams in Fig. 1B and C, and Supplementary Table 1).

Hierarchical clustering was performed for the 45 most highly expressed ISGs (ranked according to the chIFN-α-stimulated CEFs transcript list) in combined data for all microarray comparisons using the heatmap function in R (Fig. 1D). This clustering separated the samples into three main groups. The first two subsets included: (i) ISGs with high expression in all treatments but lower basal expression in untreated DF-1 cells compared with CEFs (Mx1, ISG12-2, RSAD2, LOC418700) and (ii) a single ISG (IFIT5) that is highly expressed in all conditions but relatively unchanged in untreated cells. Most ISGs fell into a third intermediary subset with expression patterns that indicate attenuated expression in DF-1 compared with CEFs for all treatments but wide-ranging basal expression levels. Amongst ISGs, SOCS1 was notable in having the most highly upregulated basal level in DF-1 (whereas it is 44^th^ in the list of ISGs ranked according to IFN-induction in CEFs). It is expressed in untreated DF-1 at levels 16-fold higher than in CEFs. Stimulation of CEFs with IFN, or by IBDV infection, raised the expression levels of SOCS1 to those equivalent to DF-1 at basal levels (Fig. 1E).

### Correlation analysis between microarray by qRT-PCR analyses

**Figure 2:**
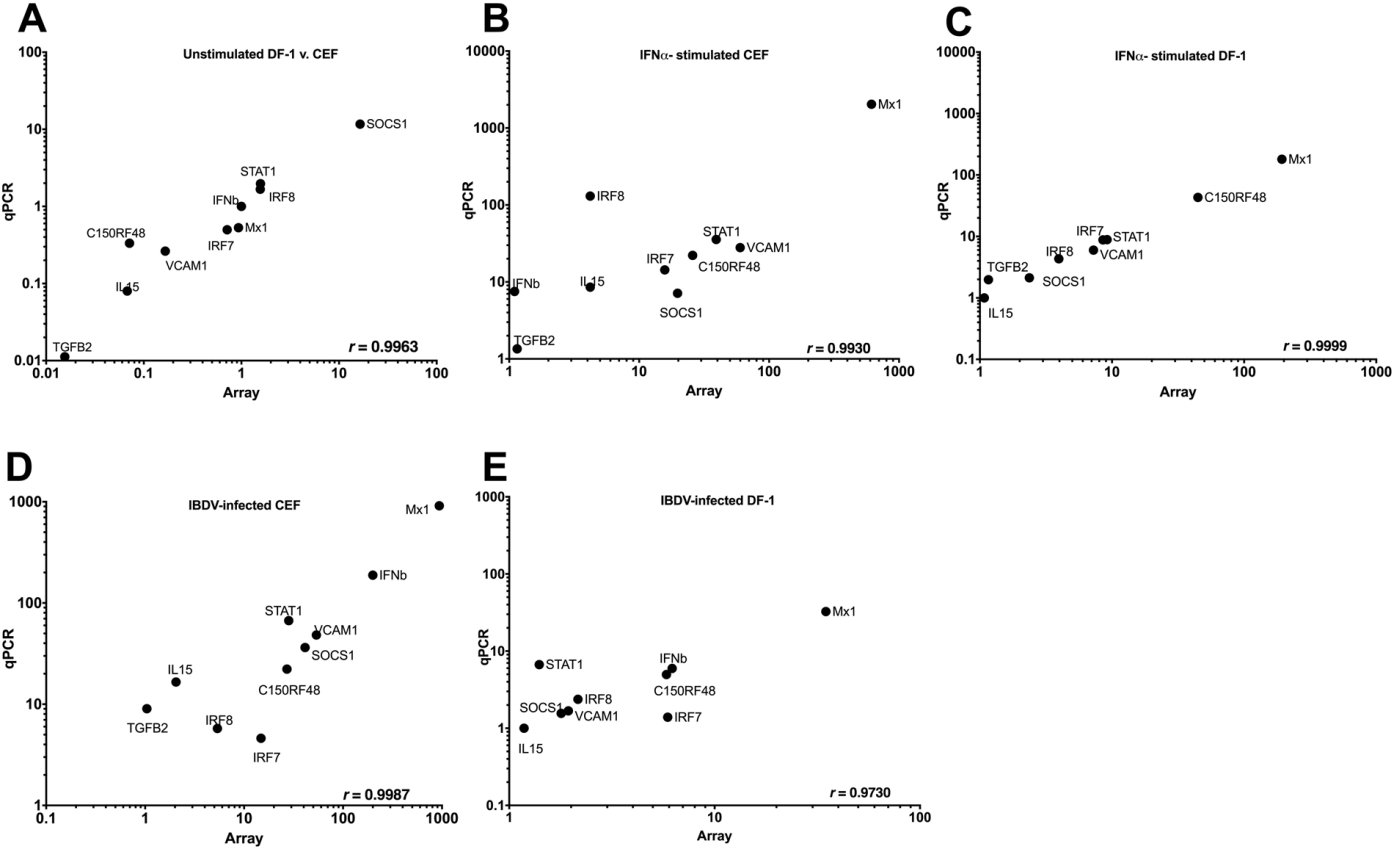
Comparison of median differential expression levels for 10 selected transcripts determined by microarray and qRT-PCR analysis. Plots show log_10_ expression fold change for the selected genes under five different conditions: (**A**) Mock-treated DF-1 versus CEFs, (**B**) chIFN-α-stimulated versus mock-treated CEFs, (**C**) chIFN-α-stimulated versus mock-treated DF-1, (**D**) IBDV-infected versus mock-treated CEFs and (**E**) IBDV-infected versus mock-treated DF-1. Pearson correlation coefficients (*r*) are shown in the lower right corner of each plot.

Microarray data were validated by quantifying the mRNA abundance of 10 selected transcripts (SOCS1, Mx1, IFN-β, TGFβ_2_, IL15, IRF7, IRF8, VCAM1, C15ORF48 and STAT1), using qRT-PCR (Fig. 2). These genes were selected among those found significantly regulated in at least one microarray comparison, and identified as significant due to their potential importance in the innate response of the cells. Pearson’s correlation test was performed to test for pairwise correlations among the two methods on all 10 genes. The correlation coefficients (*r*) for all comparisons were over 0.97, indicating the high reproducibility of results with either method. We note that IFN-β was not induced by exogenous IFN-α in either CEFs (at least as detected by microarray) or DF-1 cells but it was induced (to a lesser extent in DF-1 cells than in CEFs) by infection with IBDV, clearly demonstrating the requirement for a second signal (along with IRF3 activation) for induction of the IFN-β promoter.

### siRNA-mediated knockdown and overexpression of SOCS1 suggest that SOCS1 promotes IFN response attenuation in DF-1 cells

**Figure 3:**
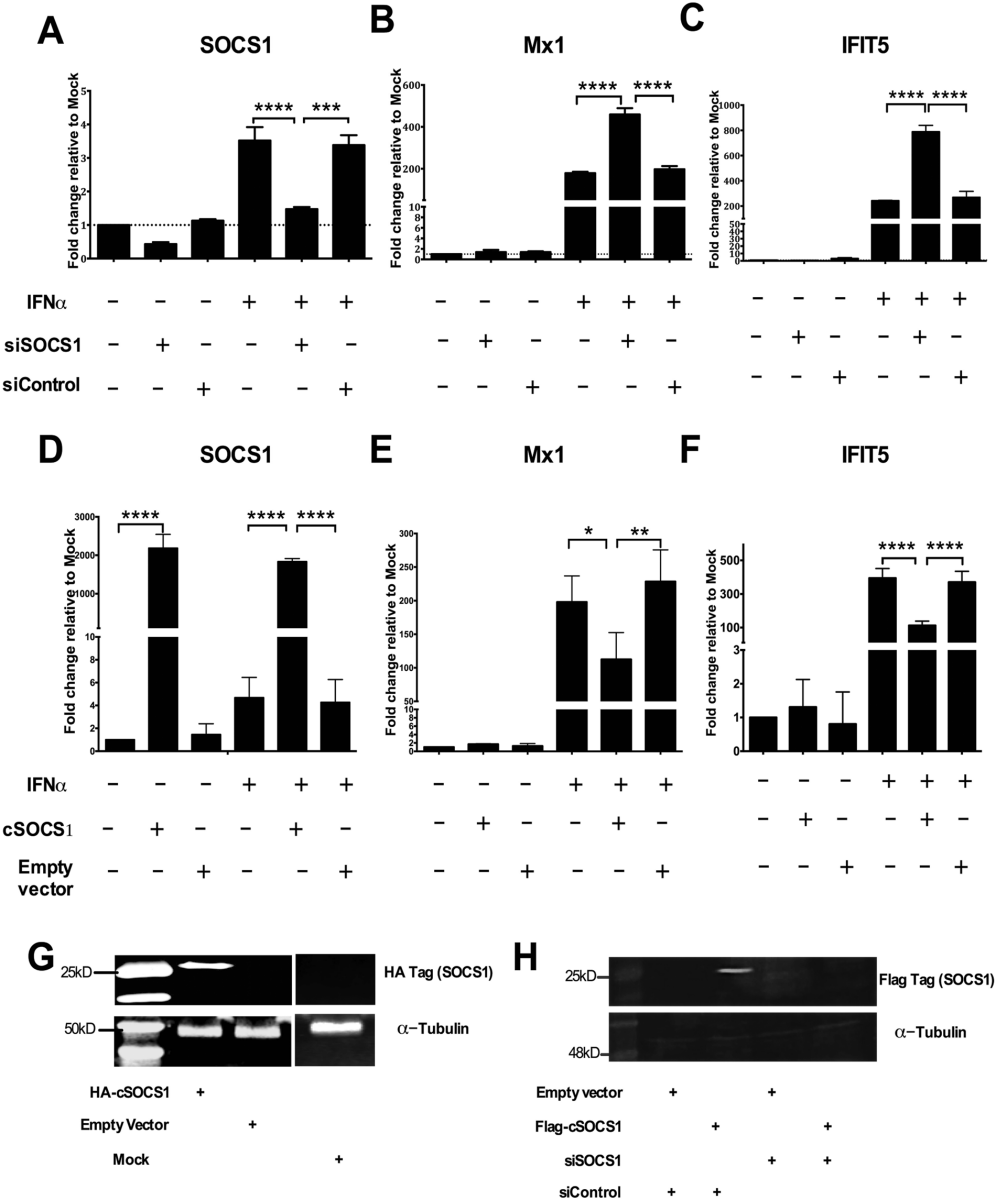
Relative suppression or induction of ISG transcription by overexpression or knockdown, respectively, of SOCS1. (**A**–**C**) DF-1 were transfected with control siRNA or siRNA specific for SOCS1 for 42 h and mock-treated or treated with chIFN-α (1000 units/ml) for 6 h. (**D**–**F**) DF-1 were transfected with either empty vector or an HA-tagged SOCS1 expression plasmid (SOCS1p) for 42 h and and mock-treated or treated with chIFN-α (1000 units/ml) for 6 h. Extracted total RNA was subjected to reverse transcription followed by quantitative PCR using specific primer sets for SOCS1 (A and D), Mx1 (B and E), IFIT5 (C and F) normalized against GAPDH (using the ΔΔCt method). Data in A-F are representative from three independent experiments. One-way (**A–F**) Anova with Bonferroni *posthoc* test were used to analyse the data. *P < 0.05, ***P < 0.001, ****P < 0.0001. (**G**) & (**H**) Immunoblots confirming expression of exogenous HA-tagged SOCS1 following transfection of DF-1 with either pcDNA4 (empty vector) or pcDNA4 expressing HA-tagged SOCS1 (**G**) and silencing of SOCS1 following transfection of DF-1 with the Flag-tagged SOCS1 construct and either siRNA for SOCS1 or a control siRNA (**H**). Panels (G) and (H) show cropped images of the immunoblots; full-length blots are presented in Supplementary Fig. S5.

To understand the effects of chicken SOCS1 on the innate response, DF-1 cells 42 h post-transfection with siRNA specific for SOCS1 (siSOCS1) or control siRNA (siControl) were stimulated with chIFN-α for a further 6 h. RNA was then collected and the expression of ISGs (SOCS1, Mx1, IFIT5) was monitored by qRT-PCR. The expression of chicken SOCS1 at 48 hours post-transfection (hpt) in cells transfected with siSOCS1 was reduced to 50% relative to that in mock DF-1 and DF-1 transfected with siControl (Fig. 3A); the siSOCS1 also efficiently limited the response of endogenous SOCS1 to chIFN-α (Fig. 3A). Knock-down efficiency of siSOCS1 was also confirmed by western blot using a Flag antibody in cell lysates from DF-1 co-transfected with siSOCS1 and a flag-tagged SOCS1 construct in pEF.pLink2 (Fig. 3H). Knocking down endogenous SOCS1 in chIFN-α-stimulated DF-1 cells led to an increase of Mx1 and IFIT5 mRNA expression compared with mock-treated cells and cells transfected with siControl (Fig. 3B and C).

Next, we over-expressed HA-tagged SOCS1, using an expression plasmid, with and without chIFN-α, and examined the expression of SOCS1, Mx1 and IFIT5 using qRT-PCR. Overexpressing SOCS1 in chIFN-α-stimulated DF-1 cells led to a significant decrease of Mx1 and IFIT5 mRNA induction compared with mock-treated cells and cells transfected with the empty plasmid (Fig. 3E and F). Western blotting confirmed the expression of the HA-tagged fusion SOCS1 protein (Fig. 3G).

### The SOCS box of chicken SOCS1 is not essential for blocking JAK/STAT signalling

As a complementary approach to demonstrating the role of SOCS1 by siRNA knockdown, we sought to abrogate its activity by deletion of known critical regions. In mammals, SOCS1 is known to interfere with the JAK-STAT signalling pathway^24^. SOCS1 is one of the 8 members of the suppressor of cytokine signalling (SOCS) and CIS family of intracellular proteins (CIS, SOCS1, SOCS2, SOCS3, SOCS4, SOCS5, SOCS6 and SOCS7)^24^. Each of these proteins has: (i) an amino-terminal domain of variable length and sequence, (ii) a kinase inhibitory region (KIR)^25^, (iii) a central SH2 domain and (iv) a carboxy-terminal 40-amino acid module known as the SOCS box^25^. The SH2 domain of SOCS1 binds to the activation loop of JAKs. SOCS1 and SOCS3 have been shown to inhibit JAK tyrosine kinase activity directly through their KIR domain^26,27^. The SOCS box interacts with elongin B and elongin C, cullins and the ring-finger domain-only protein RBX2, which recruits E2 ubiquitin transferase and mediates degradation of the proteins with which CIS-SOCS proteins associate through the SH2 domain and/or N-terminal regions^27^.

**Figure 4:**
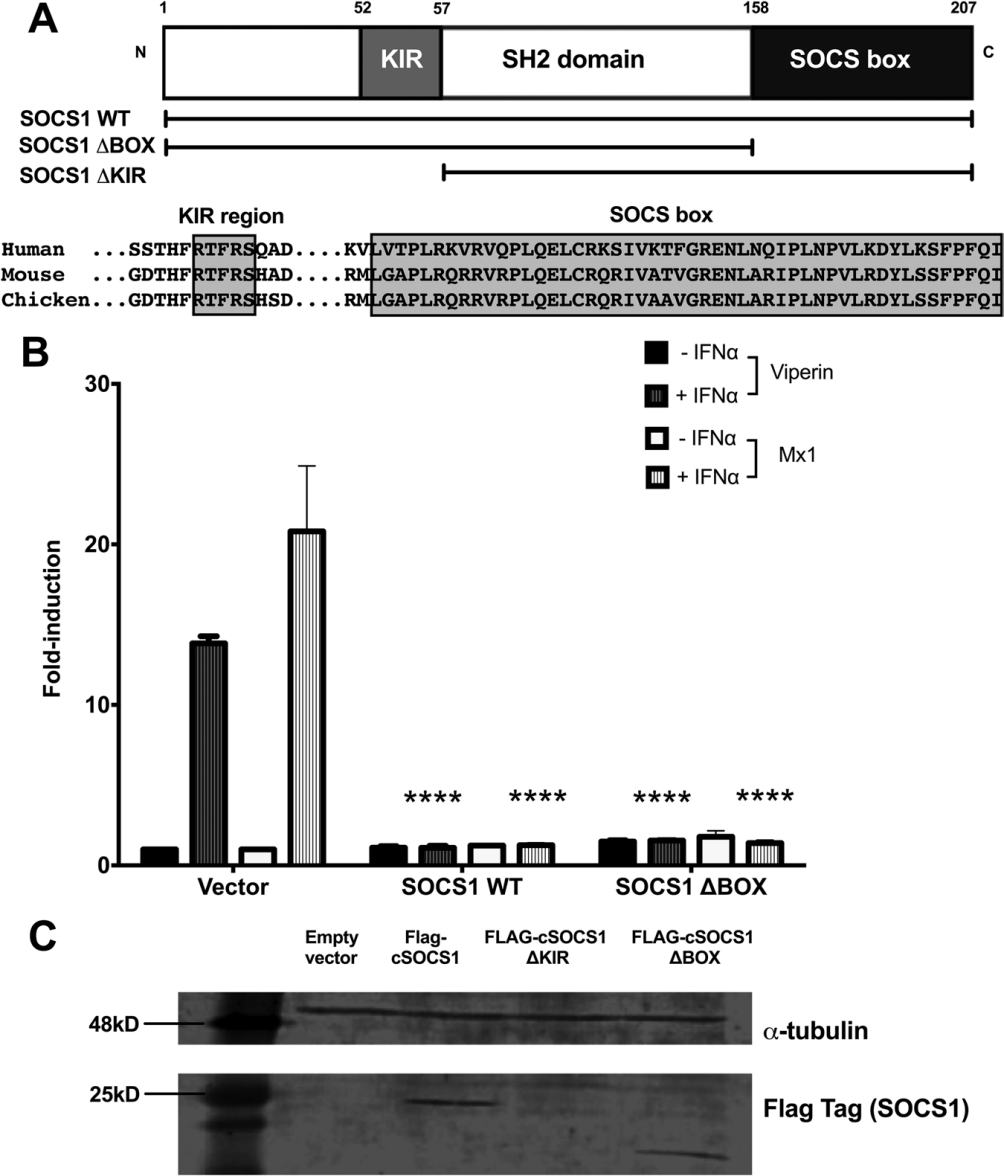
The SOCS box is not essential for ChSOCS1 inhibition of JAK/STAT signalling in DF-1. (**A**) Schematic representation of the architecture of SOCS1 protein, expression plasmids encoding wild-type (SOCS1 WT) and SOCS box deletion mutant (SOCS1 ΔBOX) and alignment of sequences (in boxes) of the KIR motif and the SOCS box domain of human, mouse and chicken SOCS1 proteins. (**B**) Luciferase reporter gene assay in DF-1 for chicken Mx1 and viperin promoters following transient transfection expression plasmids SOCS1 WT and SOCS1 ΔBOX, each with and without chIFN-α treatment. Two-way Anova with Bonferroni *posthoc* test were used to analyse the data. *P < 0.05, **P < 0.01, ***P < 0.001, ****P < 0.0001. (**C**) Immunoblot confirming expression of Flag-tag in Flag-tagged expression plasmids encoding for wild-type (WT) SOCS1 and SOCS box deletion mutant (ΔBOX). No protein was observed when DF-1 were transfected with SOCS1 KIR deletion mutant (ΔKIR) plasmid. Panel (C) shows cropped images of the immunoblots; full-length blots are presented in Supplementary Fig. S5.

We therefore generated a deletion mutant for the SOCS box region of chicken SOCS1 (Fig. 4A) and tested its ability to modulate IFN-signalling using two luciferase reporter constructs (pchMx-lucter and pchViperin-lucter) for the IFN-responsive promoters from chicken ISGs Mx1 and viperin, respectively (Fig. 4B). DF-1 cells were transiently co-transfected with pchMx-lucter or pchViperin-lucter, a beta-galactosidase reporter as well as expression plasmids for wild type SOCS1 or the SOCS1 deletion mutant, or the empty expression plasmid (pEF-FLAG.pL2). Promoter activity in cells transfected with pEF-FLAG.pL2 was induced after chIFN-α-treatment by approximately 18-fold (Mx1) and 14-fold (Viperin). Induction of the ISG promoter activity after chIFN-α-treatment was completely blocked when cells were transfected with the wild-type SOCS1. The SOCS box domain-deleted SOCS1 still blocked induction of ISG promoter activity (Fig. 4B), indicating that the SOCS box is not essential for inhibition of IFN signalling in DF-1 cells. Immunoblotting confirmed that the SOCS box-deleted SOCS1 protein was stably expressed (Fig. 4C). We also tested the effect of deletion of the kinase inhibitory region (KIR) domain. KIR-deleted SOCS1 was apparently unable to block IFN-mediated induction of ISG promoter activity (results not shown). However, immunoblotting showed that the KIR-deleted SOCS1 protein was unstable (Fig. 4C) so no conclusion can be drawn concerning whether or not the KIR domain is essential to the ability of SOCS1 to block induction of ISG promoters.

### Modulating levels of SOCS1 in CEFs and DF-1 cells affects viral yield

**Figure 5:**
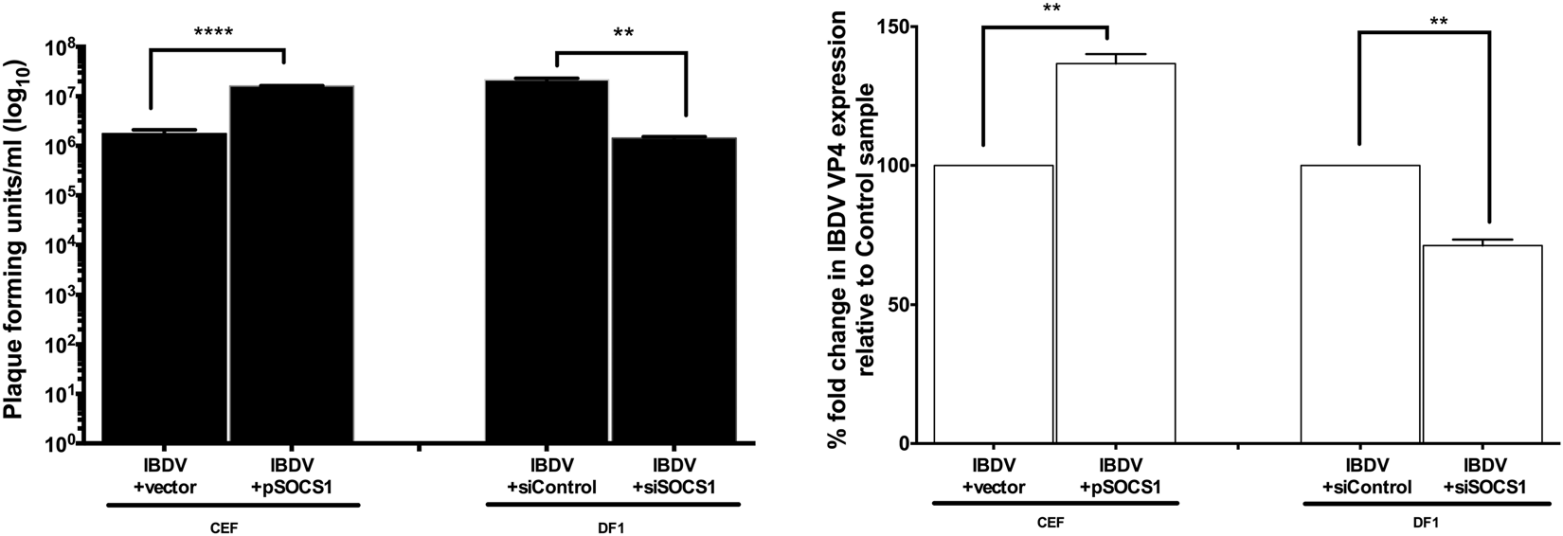
Modulating levels of SOCS1 in CEFs and DF-1 regulates viral RNA expression and virus yield. (**A**) Titres of IBDV PBG98 recovered from CEFs and DF-1 after transient transfection with a SOCS1 expression plasmid or siSOCS1, respectively. Virus titres are the sum of cell-associated and extracellular virus, determined by plaque assay on CEFs (Mean ± SEM). (**B**) Fold change (percent) of IBDV VP4 RNA levels in CEFs and DF-1 after transient transfection with a SOCS1 expression plasmid or siSOCS1, respectively. Virus titres and viral RNA levels were compared with those from cells transfected with empty vectors or control siRNA, as appropriate. Data are representative from three independent experiments. An unpaired t-test with Welch’s correction (Two-tailed) was used to analyse the data. **P < 0.01, ****P < 0.0001.

Induction of ISG expression by chIFN-α is reduced by overexpression of SOCS1; conversely, SOCS1 knockdown reverses the block to ISG induction. To determine whether modulation of SOCS1 expression could therefore affect virus replication, the titres of IBDV PBG98 at 16 h p.i. were determined for CEFs and DF-1 cells in which SOCS1 had been overexpressed or knocked down, respectively (Fig. 5A). Overexpression of exogenous SOCS1 in CEFs resulted in 1 log increase in IBDV titre (pfu/ml) compared with CEFs transfected with an empty vector. Conversely, knock down of endogenous SOCS1 levels in DF-1 cells resulted in a corresponding decrease of viral titre (approximately 1 log) at 16 h p.i. compared with DF-1 cells transfected with control siRNA (Fig. 5A).

The transfection efficiency of siSOCS1 in CEF primary cells was too low to result in significant downregulation of endogenous SOCS1. Conversely, transient overexpression of SOCS1 in DF-1 cells (which already have a high constitutive expression of SOCS1) did not result in significant increase in the total levels of SOCS1. To overcome the latter issue, we generated a new, DF-1-derived cell line stably overexpressing SOCS1 tagged with V5. We found that replication of IBDV is improved by approximately 1 log compared with control (expressing the empty vector) or parental DF-1 cells (data not shown).

Consistent with the differences in viral titre, qRT-PCR showed 37% increase in IBDV polyprotein (VP4) mRNA levels following overexpression of exogenous SOCS1 in CEFs but 29% decrease following knock down of endogenous SOCS1 in DF-1 cells (Fig. 5B). Western blot analysis of the effects of overexpression of SOCS1 in DF-1 cells infected with IBDV (Supplementary Figure S2) also demonstrated elevated levels of the IBDV VP2/3 protein but reduced levels of phosphorylated STAT1 compared to untransfected control cells (total levels of STAT1 were unchanged), indicating partial inhibition of virus-induced activation of STAT1 by SOCS1.

### Regulation of SOCS1 in DF-1 cells

SOCS1 basal expression in DF-1 cells might be upregulated by specific transcription factors, distinct from those involved in induction of ISGs. Bioinformatic analysis identified an overrepresentation of CREB1 transcription factor binding sites in the promoters of genes upregulated in DF-1 cells relative to CEFs and, interestingly, a CREB1 binding site (TGACGTCA) is located in the promoter proximal region of chicken SOCS1 extending to within 2 bases of the transcription-starting site (Supplementary Table 3). CREB1 is highly conserved in 18 bird species (data not shown) and in mammals mediates the transcription of genes containing a cAMP-responsive element, including the inflammation-related: IL-2, IL-6, IL-10, and TNF-α^28^. As well as containing a CREB1 binding site, the chicken SOCS1 promoter region contains a complex array of potential regulatory elements that await functional investigation.

Although direct associations of transcription factors are considered an important mechanism to regulate gene expression, we cannot rule out other mechanisms, such as epigenetic modifications, that might independently or synergistically activate basal expression levels of chicken SOCS1 in DF-1 cells. Epigenetic modifications have increasingly been associated with activation or repression of innate signalling^29^. These chromatin modifications determine how tightly DNA is wound around the histones and usually involve up- or down-regulation of histone acetylases/deacetylases or methyltransferases, leading to the activation of specific gene expression pathways, including innate signalling^29,30^. Histone acetylation, deacetylation, and hypermethylation have been previously reported to regulate SOCS1 expression in cancer cells^31^. Analysis of microarray data presented here showed that the histone deacetylases (HDAC) 9 and 11 (Supplementary Table 1) were downregulated in untreated DF-1 compared with CEFs. Addition of the HDAC inhibitor sodium butyrate (SB; 2 mM) to cell culture medium induced significantly the expression of SOCS1 in uninfected and IBDV-infected CEFs, while it had no effect on uninfected or IBDV-infected DF-1 cells (Supplementary Fig. S1). Addition of SB to IBDV-infected CEFs increased expression levels of ISGs such as IFIT5 (significantly) and MDA5, IRF7, STAT1 and Mx1 (not significantly) (Supplementary Fig. S1).

## Discussion

Infection studies in chicken cell lines are compromised by a lack of definitive understanding of the chicken innate response and in particular the type I IFN response, which is the first line of defence, particularly upon virus infection. Despite the high degree of evolutionary conservation, and assumed similarity in overall function, there are significant differences between the innate response gene repertoires of chicken (and birds in general) and mammals^32,33^. In birds, as in mammals, interferons trigger tyrosine phosphorylation and activation of members of the Janus kinase (JAK) family of cytoplasmic tyrosine kinases that activate the phosphorylation of signal transducers and activator of transcription (STAT)1 and STAT2, though the latter has only recently been identified in birds and mapped to chicken chromosome 33 (unpublished and Galgal5)^34^. Phosphorylated STATs undergo dimerization and associate with factors such as IFN-regulatory factor 9 (IRF9), though this has not yet been identified or characterised in birds, to form the IFN-stimulated gene factor 3 (ISGF3)^35,36^. This complex then translocates to the nucleus and binds to IFN-stimulated response elements (ISREs) in DNA to activate the transcription of hundreds of IFN-stimulated genes (ISGs), which mediate various important biological processes in the cell including antiviral and other innate response functions^22,34,36^.

In a separate study, we have characterised the transcriptomic response of CEFs to recombinant chIFN-α, as determined by RNA-seq and two separate microarray technologies, establishing a catalogue of ISGs that can be compared to those induced by other inducers (such as dsRNA), by virus infection or in different cell types^4^. Here, we have evaluated the type I IFN response of DF-1 cells in comparison with that of CEFs, based on microarray data validated by subsequent qRT-PCR. These show that recombinant chIFN-α (or infection with an attenuated IBDV strain) can induce a relatively broad range of chicken ISGs in DF-1 cells. However, the number of ISGs induced and the levels of their induction were much lower in DF-1 cells compared with CEFs. Further evidence from RNA-seq data (unpublished) shows lower basal levels for several ISGs, such as Mx1, IL15, IL1R1 and IFITM3 (data not shown), in DF-1 cells compared with CEFs. This inherently weakened antiviral IFN response might, in part at least, explain the apparently improved ability of DF-1 cells, compared with CEFs, to propagate several viruses.

Though perhaps less relevant in culture, it is noteworthy that the basal level of proinflammatory gene expression was also lower in untreated DF-1 cells compared to CEFs. Genes encoding proinflammatory cytokines (IL6, IL8L2), chemokines (CxCL14) and proinflammatory mediators (PTGS2) are downregulated in DF-1 cells, as also reported by Kong *et al*.^18^.

The microarray data presented in this study, conducted using the 35 K Affymetrix Chicken Genome Array, are consistent with data from a previously reported comparison between untreated DF-1 and CEFs conducted with the 44 K 60-mer oligonucleotide Agilent chicken microarrays by Kong *et al*.^18^. Both studies demonstrated that DF-1 cells exhibit a suppression of cell death pathways, altered mitochondrial related gene expression and enhanced capacity for molecular transport. Upregulation of cell cycle regulatory factors (p53, E2F1, the CDKs and cyclins) and of c-src in DF-1 cells probably reflects their higher replication rate compared with CEFs. Our results showed preferential activation of genes involved in metaphase and G2-M DNA damage checkpoints in DF-1 cells, which might be associated with their immortalized phenotype.

Here we show that the chicken ISG SOCS1, a negative regulator of cytokine signalling in mammals, is implicated in the innate response and proinflammatory phenotypes of DF-1 cells, where it might also play a role in their replication and immortal phenotypes. The basal level of expression of chicken SOCS1 is 16-fold higher in DF-1 cells than in CEFs, as previously reported by Kong *et al*.^18^. Like its mammalian counterparts, chicken SOCS1 is a relatively small protein of 207 amino acids (23kD); it shares high amino acid sequence identity (~65%) with mammalian orthologues. Transient overexpression of chicken SOCS1 in IFN-stimulated DF-1 led to a significant relative decrease in the expression of Mx1 and IFIT5; conversely, knockdown of endogenous SOCS1 increased their induction. Moreover, in a SOCS1-overexpressing derivative of DF-1, the IFN-mediated induction of ISGs Mx1 and viperin was blocked. Like mammalian SOCS1, therefore, chicken SOCS1 acts as a robust attenuator of IFN signalling to block ISG induction.

Although the SOCS box is generally essential for the full inhibition of IFN signalling by mammalian SOCS proteins, our results demonstrate that it is not vital for the ability of chicken SOCS1 to abrogate IFN signalling and subsequent induction of ISGs. In mammals, the SOCS box interacts with the ubiquitin ligase complex promoting proteasomal degradation of proteins it targets. It is possible that ubiquitin-mediated, proteasomal degradation of JAKs is not essential to the inhibitory mechanism(s) of SOCS1 in the chicken, which might, therefore, reflect different modes of interaction of SOCS1 with JAKs and overall regulation of JAK/STAT signalling.

The biological function of SOCS1 extends well beyond its regulatory role in the antiviral IFN response. It regulates a wide range of immunological processes including dendritic cell functions^37^, T-cell differentiation^38^, class I MHC mediated antigen processing and presentation^39^ and IFN-γ signalling^38,40^. The observation that SOCS1 attenuates the IFN response in pluripotent human cells suggests that it plays an important role(s) in differentiation and development^41^. SOCS1 also interacts directly with and activates the p53 tumour suppressor through its SH2 domain, thereby regulating the process of oncogene-induced senescence^42^.

SOCS1 is able to enter the nucleus and interact with the p65 subunit, thereby inhibiting the nuclear NF-κB signalling pathway^43^ and inflammatory signalling pathways involving IL6^40^, IL2^44,45^ and the TLR signalling cascades^45^. This might explain the suppression of basal level expression of proinflammatory cytokines we observed in untreated DF-1 cells compared to CEFs.

Based on the downregulation of the histone deacetylases that we observed in DF-1 cells relative to CEFs and the induction of SOCS1 expression in CEFs treated with HDAC inhibitor, it is plausible that histone deacetylation in CEFs, and its downregulation in DF-1, offers a mechanism by which SOCS1 expression might be suppressed in CEFs and become enhanced in DF-1. We cannot rule out the role of other epigenetic mechanisms influencing basal SOCS1 expression (in either CEFs or DF-1). Nor, without extensive promoter analysis, can we eliminate the possibility that mutation(s) in promoter or enhancer elements affect basal SOCS1 expression in DF-1 cells.

It is possible that reprogramming events during spontaneous immortalization of DF-1 from CEFs might have indirectly activated SOCS1. It is equally plausible, however, that its overexpression was selected, possibly to suppress aberrant signalling leading to apoptosis. In this context, it is interesting to note (Fig. 1D) the lower basal level of expression of some ISGs (such as MX1, RSAD2 and ISG12-2) in DF-1 cells compared to CEFs. Although this could be indicative of other mechanisms in play in addition to elevated constitutive SOCS1, there is now recognition that low-level constitutive IFN can promote “tonic signalling” to activate STAT1^46,47^. Modulation of such “tonic signalling” by the elevated constitutive levels of SOCS1 in DF-1 cells might conceivably account for the suppressed basal levels of some ISGs in DF-1 cells.

Overexpression of SOCS1 has been reported to have a proviral effect in infection and replication for viruses such as HCV, herpes simplex virus 1 (HSV-1) and vaccinia virus^48–50^. A recent study has also used a peptide, pJAK2(1001–1013), which corresponds to the activation loop of JAK2, as a SOCS1 antagonist. The antagonist enhanced innate and adaptive immune responses against a broad range of viruses including HSV-1, vaccinia virus, and encephalomyocarditis virus^51^. In this study we used PBG98, an attenuated vaccine strain of IBDV, as a model avian pathogen^52^. We found that decreasing the elevated levels of SOCS1 in DF-1 cells inhibited IBDV transcription and replication. Conversely, increasing the levels of SOCS1 in CEFs restored the replication rate and viral yield to levels that are normally observed in DF-1.

Altering the host innate response via modulating intracellular SOCS1 levels might offer a simple and flexible solution to enhance viral propagation in culture, especially using IFN-sensitive viruses, with potential applications in diagnosis and vaccine production.

Here we have examined the interplay between SOCS1 and the chicken IFN antiviral response in the context of stimulation with recombinant IFN or IBDV infection, improving our understanding of DF-1 cells, which are becoming increasingly popular in studies of avian infection and innate immunity. Several mammalian cell lines such Vero, HuH7.5 and BHK21, are known to be defective, by various mechanisms, in intrinsic innate immunity and thereby more permissive to virus infection^53–55^. DF-1 therefore provide another example of IFN insufficiency, perhaps suggesting that this phenotype is beneficial to the establishment of such cell lines. Those studying avian innate responses in DF-1 cells, as with mammalian cells in which IFN deficiencies are better known, need to be aware of potentially confounding effects due, at least in part, to their higher basal expression of SOCS1.

## Methods

### Cell culture

Primary CEFs, prepared by trypsin/EDTA dissociation of 10-day-old chicken embryos^56–58^, were provided by the Institute for Animal Health (now The Pirbright Institute) from their Compton Laboratory, Berkshire, UK. They were seeded in T25 flasks (Greiner Bio One; 5.6 × 10^6^ cells/flask) and cultured overnight in 5.5 ml 199 media (Gibco, Invitrogen) supplemented with 8% heat-inactivated newborn bovine serum (NBCS; Gibco, Invitrogen), 10% tryptose phosphate broth (TPB; Sigma), 2% nystatin (Sigma) and 0.1% penicillin and streptomycin (Gibco, Invitrogen). DF-1 were propagated in Dulbecco’s minimal essential medium (DMEM) (Life Technologies) supplemented with 10% heat-inactivated fetal bovine serum (Life Technologies) and penicillin and streptomycin. All cells were incubated at 37 °C and 5% CO_2_. For sodium butyrate experiments, sodium butyrate (0.5–2.0 mM; Sigma-Aldrich) was added directly to cells in 6-well plates and optimization experiments were carried out for 18 hours in both CEFs and DF-1 cells. Samples for the mock controls were treated with DMEM.

### Plasmids and luciferase reporter gene

SOCS1 expression plasmid was constructed by cloning the full-length coding region of chicken SOCS1 gene using *Hin*dIII and *Not*I cloning sites into the eukaryotic expression vector pcDNA4, that had been modified to encode an N-terminal influenza hemagglutinin (HA) epitope tag. The plasmid DNA was purified from transformed bacteria and concentration determined by UV spectroscopy. For luciferase reporter assays, the promoters from chicken ISGs Mx and viperin (RSAD2) were amplified from CEF genomic DNA using Accuprime Pfx DNA polymerase (Invitrogen). The promoter region amplified for Mx had been described previously^59^; primers contained *Bgl*II and *Mlu*I sites. The region amplified for viperin was −343 to −193 relative to the ATG start codon; primers contained *Bam*HI and *Mlu*I sites. Both promoter fragments were inserted between the *Bam*HI and *Mlu*I sites of ptkΔ(− 39)lucter^60^ producing pchMx-lucter and pchviperin-lucter. Full-length chicken SOCS1 was also amplified from RNA using Q5 High-Fidelity DNA polymerase (New England Biolabs, USA) and primers containing *Nco*I and *Eco*RI and was cloned into pEF.pLink2^61^. SOCS1 ΔKIR was produced by amplifying the region 235–624 of SOCS1, whilst SOCS1 ΔSOCSBox was produced by amplifying the region 1–495. Both fragments used *Nco*I and *Eco*RI for cloning into pEF.pLink2. All clones were verified by sequencing.

### Virus infections and chIFN-α stimulation

For virus infection studies, the attenuated IBDV vaccine strain PBG98 (propagated in CEFs^52^), a strong inducer of innate responses^62^, was used to stimulate cells. Viral titres were determined by classical plaque assay in CEFs. Fully confluent DF-1 and CEFs grown in T25 flasks were washed with phosphate- buffered saline (PBS) and infected for 2 h with IBDV (MOI 5) or mock-infected. The inoculum was then removed and cells were washed and further incubated in maintenance medium (2% fetal bovine serum) for 14 h until determination of viral titre. For chIFN-α stimulation experiments, recombinant chicken chIFN-α was prepared as previously reported^63^ and was added in culture media to a final concentration of 1000 U/ml. Confluent cells were treated with chIFN-α or mock treated and incubated for six hours before harvesting. Cells were stored at −80 °C in RNAlater (Sigma) until RNA extraction.

### SOCS1 siRNA knockdown and overexpression

For both siRNA knockdown and overexpression experiments, DF-1 were seeded overnight at 4.5 × 10^6^ cells per plate in a 6-well plate to achieve 50% confluency. Cells were transfected with 50 nM of siRNA (designed and supplied by Sigma-Aldrich) coupled with JetPrime transfection reagent (Polyplus Transfection SA, Illkirch, France) for 48 h according to manufacturer’s instruction with or without a 6 h 1000 units/ml chIFN-α treatment. RNA and protein samples were obtained from the cells using standard techniques. Knockdown efficiency of siRNA for SOCS1 was determined at 48 h post transfection with qRT-PCR and western blot. Sense (5′-CGCAGAAGAAUUGUUUCUU[dT][dT]-3′) or antisense (5′-AAGAAACAAUUCUUCUGCG[dT][dT]-3′) siRNA were used to target chicken SOCS1. Sense (5′-CGCAGAAGUUAUGUUUCUU[dT][dT]-3′) or antisense (5′-AAGAAACAUAACUUCUGCG[dT][dT]-3′) siRNA, in which bases 9 through 11 (underlined) were replaced with their complement^64^, were also used as controls.

To investigate the possibility of “off target” effects for siSOCS1, BLAST analysis (Supplementary Fig. S3) of siSOCS1 sequence was conducted, showing that only SOCS1 returned 100% identity for 19 contiguous bases. The next best chicken hits (the only two to achieve 100% identity for 15 contiguous bases) were nuclear assembly factor 1 ribonucleoprotein (NAF1) and ADP-ribosylation factor GTPase activating protein 3 (ARFGAP3). “Off target” effects of siSOCS1 on expression of these two genes were assessed by treating DF-1 cells, 42 h post-transfection (or mock-transfection) with siSOCS1, with chIFN-α for a further 6 h, or by infecting with PBG98 for 16 h, as described above. RNA was then collected and the expression of SOCS1, NAF1 and AFRGAP3 was monitored by qRT-PCR. siSOCS1 reduced expression of SOCS1 by about 50% relative to mock-transfected but no reduction in the expression of NAF1 or ARFGAP3 was observed (Supplementary Fig. S4), indicating good specificity for siSOCS1.

For overexpression of SOCS1, a pcDNA4/HA/SOCS1 expression plasmid was used, as described above. Cells were transfected with 1 μg of a SOCS1 expression vector using the jetPrime transfection reagent (Polyplus Transfection SA, Illkirch, France) according to manufacturers instructions for 48 hours at 37 °C and 5% CO_2_. Samples for the mock controls were transfected with an empty control plasmid (pcDNA4). 6 h treatment of cells with chicken IFN-α was used to stimulate cells before collection of samples for qRT-PCR analysis. For co-transfections, the SOCS1 expression vector (0.25 μg) was combined with each siRNA as per the manufacturer’s recommendations. After 48 h, RNA and protein samples were collected for follow-up studies.

### Luciferase assay

DF-1 in 12-well plates were transfected with: Mx or viperin promoter reporters (100 ng), the constitutive reporter plasmid pJATlacZ (100 ng) and either plasmids driving the overexpression of SOCS1 wild-type or SOCSbox deletion mutant or the control empty vector pEFPlink2 (200 ng). Following recovery for 24 h, cells were either left untreated or treated with 1000 units/ml recombinant chIFN-α treatment and incubated for 6 h. Luciferase assays were carried out, and data were normalized using β-galactosidase measurements and expressed as fold induction over control.

### Western blot

Proteins for western blots were harvested from DF-1 cultured in T75 flasks. CelLytic-M solution (Sigma-Aldrich; 600 μl) was added to the cell pellet, and the supernatant was collected after centrifugation at 15,000xg for 15 minutes before protease inhibitor (Roche) was added. To every 20 μl of sample, 5 μl of 4X loading buffer (Bio-Rad) was added, and the samples were heated at 60 °C for 5 minutes. They were then separated on a 12% sodium dodecylsulfate polyacrylamide gel, alongside a protein ladder (Precision Plus Protein Dual Colour Standards, Bio-Rad). Samples (20 μg) were loaded for each well, and the gel was run at 150 V for 2 hours. The proteins were then electro-transferred to nitrocellulose membranes (Hybond-C extra, Amersham Life Science) at 100 V for 1 hour, and blocking was carried out using 5% milk (Sigma-Aldrich) in PBS containing 0.1% Tween-20 (Sigma-Aldrich) for 2 hours. After washing with PBS for five times, the membranes were either incubated with mouse monoclonal anti-FLAG (M2) (Sigma-Aldrich), rabbit monoclonal anti-HA (Sigma-Aldrich), or rabbit monoclonal α-tubulin (Cell signalling Technology) antibodies overnight at 4 °C with gentle agitation. The membranes were then washed five times with PBS, and incubated with goat anti-rabbit or donkey anti-mouse secondary antibodies (LI-COR) in the dark for 1 hour. Scanning was then carried out using the Odyssey Imaging system (LI-COR). Images of full-size immunoblots are shown in Supplementary Information (Supplementary Fig. S5).

### RNA extraction and processing of samples for microarray analysis

Total RNA was extracted from mock-, infected-, and IFN-stimulated DF-1 and CEFs using an RNeasy kit (Qiagen) according to the manufacturer’s instructions. On-column DNA digestion was performed using RNase-free DNase (Qiagen) to remove contaminating genomic DNA. RNA samples were quantified using a Nanodrop Spectrophotometer (Thermo Scientific) and checked for quality using a 2100 Bioanalyzer (Agilent Technologies). All RNA samples had an RNA integrity number (RIN) ≥9.6. RNA samples were processed for microarray using the GeneChip® 3′ IVT Express Kit (Affymetrix) following the manufacturer’s instructions. Total RNA (100ng) was used as input and quality checks were performed using the Bioanalyzer at all stages suggested by the manufacturer. RNA samples were processed in batches of 12 but batch mixing was used at every stage to avoid creating experimental bias. Hybridisation of RNA to chips and scanning of arrays was performed by the Medical Research Council’s Clinical Sciences Centre (CSC) Genomics Laboratory, Hammersmith Hospital, London, UK. RNA was hybridised to GeneChip Chicken Genome Array chips (Affymetrix) in a GeneChip Hybridization Oven (Affymetrix), the chips were stained and washed on a GeneChip Fluidics Station 450 (Affymetrix), and the arrays were scanned in a GeneChip Scanner 3000 7 G with autoloader (Affymetrix).

### Microarray data analysis

A two-way ANOVA (variables: cell type and treatment) adjusted with the Benjamini–Hochberg multiple-testing correction (false discovery rate (FDR) of *P* < 0.05) was performed with Partek Genomics Suite (v6.6, Partek) across all samples. Principal component analysis confirmed that batch mixing had prevented introduction of experimental bias. Comparisons were conducted between treated cells (IFN-stimulated or IBDV-infected) versus mock-treated cells for each cell line (CEFs or DF-1) and between untreated DF-1 versus CEFs. The analysis cut off criteria were fold change ≥ ± 3.0 and *P*-value ≤ 0.01. The Affymetrix chicken genome arrays contain probe sets for detecting transcripts from 17 avian viruses, including IBDV, allowing confirmation of viral infection.

Data mining and enrichment analysis was performed using the MetaCore software suite (Clarivate Analytics, https://clarivate.com/products/metacore). Enrichment analysis consisted mapping gene IDs of the datasets onto gene IDs of human orthologues in entities of built-in functional ontologies represented in MetaCore by pathway maps and process networks. Statistical significance was measured by the number of genes that map onto a given pathway and was calculated on the basis of p-value, based on hypergeometric distribution (a built-in feature of MetaCore). Full enrichment analysis for the untreated DF-1 versus CEFs dataset (enrichment by gene ontology (GO) processes, process networks, pathway maps and protein function) as well as a list of transcription factors identified using the Metacore transcription regulation algorithm is presented in Supplementary Table 2.

Visualisation of gene expression data was conducted with GeneSpring GX (v.13.1, Agilent Technologies); GO search and grouping was peformed using the PANTHER classification system (http://www.pantherdb.org/). Fold change values for the top 45 ISGs identified in IFN-stimulated CEFs were displayed in a heat map in combined data from all microarray comparisons (Hierarchical Clustering) generated using the ggplot2 package within the open source R console (3.1.1). Original microarray data produced or used in this study have been deposited according to the MIAME guidelines in the public database ArrayExpress (http://www.ebi.ac.uk/microarray-as/ae/) (Acc. No: E-MTAB-4028). That entry includes six CEL files, used for meta-analysis of CEFs plus or minus IFN as controls for DF-1, originally deposited and available as E-MTAB-3711, as described previously^4^. Tables showing ArrayExpress information for the CEL files in each entry are presented as an EXCEL workbook in Supplementary Table 5.

*De novo* motif prediction analysis was conducted using the R console (3.1.1) and the JASPAR2014 (1.1.1) and TFBSTools (1.4.0) packages. Motifs obtained from databases have been sorted by match score. Minimum scores of 80 and 90% have been considered as valid (Supplementary Fig. S1).

### Quantitative real-time RT PCR

Quantitative real-time RT PCR was performed on RNA samples using a two-step procedure. RNA was first reverse-transcribed into cDNA using the QuantiTect Reverse Transcription Kit (Qiagen) according to manufacturer’s instructions. qPCR was then conducted on the cDNA in a 384-well plate with a ABI-7900HT Fast qPCR system (Applied Biosystems). Mesa Green qPCR MasterMix (Eurogentec) was added to the cDNA (5 μl for every 2 μl of cDNA). The following amplification conditions were used: 95 °C for 5 minutes; 40 cycles of 95 °C for 15 seconds, 57 °C for 20 seconds, and 72 °C for 20 seconds; 95 °C for 15 seconds; 60 °C for 15 seconds; and 95 °C for 15 seconds. Primer sequences for genes that were used in the study are given in Supplementary Table 4. The output Ct values and dissociation curves were analysed using SDS v2.3 and RQ Manager v1.2 (Applied Biosystems). Gene expression data were normalized against the housekeeping gene GAPDH, and compared with the mock controls using the comparative C_T_ method (also referred to as the 2^−ΔΔCT^ method^65^). All samples were loaded in triplicate.

### Statistical analysis

To determine the significance of differences between experimental groups, one-way ANOVA t-tests were performed using the fold change scores with a Bonferroni multiple comparisons test or unpaired t tests with Welch’s correction. *P*-values were set at 0.05 (*P* ≤ 0.05) unless indicated otherwise. Error bars represent the standard error of the mean (SE). The correlation of expression values between microarray analysis and qRT-PCR was statistically assessed by calculation of Pearson’s correlation coefficient using the built-in function of GraphPad Prism (v.6.0).

### Data Availability

The datasets (Acc. No: E-MTAB-4028) generated and analysed during the current study are available in the EMBL-EBI ArrayExpress repository (https://www.ebi.ac.uk/arrayexpress/experiments/E-MTAB-4028/; released 15 January 2016; as described in Supplementary Table 5). All other data generated or analysed during this study are included in this published article (and its Supplementary Information files) or are available from the corresponding author on reasonable request.

## Electronic supplementary material

Supplementary Information

Supplementary Table 1

Supplementary Table 2

Supplementary Table 3

Supplementary Table 5
