# Supplementary material for: Constitutively elevated levels of SOCS1 suppress innate responses in DF-1 immortalised chicken fibroblast cells
Source: Sci Rep. 2017 Dec 13;7:17485. doi: 10.1038/s41598-017-17730-2 (PMC5727488; doi:10.1038/s41598-017-17730-2)
Supplement: Supplementary file 1 — Supplementary Information [file 41598_2017_17730_MOESM1_ESM.pdf]

Supplementary Info

Constitutively elevated levels of SOCS1 suppress innate responses in DF-1 immortalised chicken fibroblast cells

Efstathios S. Giotis
Craig S. Ross
Rebecca C. Robey
Axel Nohturfft
Stephen Goodbourn
Michael A. Skinner

Supplementary Table 1: Excel workbook file (.XLS) with 5 spreadsheets showing **Full lists of differentially regulated genes determined by 5 comparisons of 6 different conditions analysed by microarray, as summarised in Fig. 1.** Each individual tab shows one of the following comparisons: (a) chIFN-α stimulated versus mock-treated CEFs, (b) chIFN-α stimulated versus mock-treated DF-1, (c) IBDV-infected versus mock-treated CEFs, (d) IBDV-infected versus mock-treated DF-1 and (e) mock-treated DF-1 versus mock-treated CEFs**.** Headings are: Probeset ID, Gene Symbol, Gene Title, RefSeq Transcript ID, p-value (FDR) and Fold-change.

Supplementary Table 2: Excel workbook file (.XLS) with spreadsheets showing **gene set enrichment analysis (using MetaCore) for comparison between untreated DF-1 and CEFs.** MetaCore (Clarivate Analytics) was used to perform gene set enrichment analysis of the differentially regulated genes with functional Gene Ontologies (GO) and biological processes. The probability of a random intersection between a set of genes with ontology processes was estimated with the "P" value of the hypergeometric intersection. Each individual tab shows one of the following analyses: (a) enrichment by pathway maps, (b) enrichment by process networks, (c) enrichment by GO processes, and (d) enrichment by protein function as well as (e) a list of transcription factors whose targets are overrepresented in the differentially expressed gene list. Keys to column headings for tabs (d) and (e) appear in columns L-M and M-N, respectively.

Supplementary Table 3: Excel spreadsheet workbook file (.XLS) with worksheets showing results of **Motif-Search Analysis of the Chicken SOCS1 Promoter** (with minimum scores of 80 and 90%) showing the coordinates of the transcription factor binding sites (TFBSs) within the promoter (SOCS1p) and statistical significance (score). The "+" and "−" signs within brackets indicate the strand orientation of the TFBS.

Supplementary Table 4: Table showing real-time quantitative RT-PCR primers.

mRNA target	NCBI accession	Forward primer (5' to 3')	Reverse primer (5' to 3')
**GAPDH**	NM_204305.1	GGCACTGTCAAGGCTGAGAA	TGCATCTGCCCATTTGATGT
**IFITM3**	XM_001233949.3	CTATTCCCATCTCTCCGCAGC	AGATCTTCGCTGTCCTCCCA
**IFIT5**	XM_421662.4	TGCTTCACCAGCTAGGACTCTGC	TGGCTTTTGCTCTGTCACCACTTTG
**IFN-α**	EU367971.1	CCACCGCTACACCCAGCACC	ATGGCGAGGTGAAGGTTGCGA
**IFN-β**	GU119897.1	CAGTCTCCAGGGATGCACAG	GAGAAGGTGGTGGTGAGAGC
**IL15**	NM_204571.1	CACTGTAAGTGGTCAGACGTTCTGA	GGTTCCTGGCATTCTATATCCTCGT
**IRF7**	NM_205372.1	CCATCCCCGGCTGAGGAGCT	CAGCGGCTGTCGTCCACCTG
**IRF8**	NM_205416.1	AGCCCCTCTTTGCCCCTGGT	GCTGAGGGACAGCCTGCAGC
**ISG12-2**	NM_001001296.5	TGACCAGAACGTCCACAAAGCCG	ACCTGCTCCTGGACCGATGCTT
**MDA5**	NM_001193638	AAGATGAAGCAGAGGGCAGA	CCACTGCCTGTAGGGAGACA
**MX1**	NM_204609	CACACCCAACTGTCAGCGAT	ATGTCCGAAACTCTCTGCGG
**RSAD2**	XM_426208	GGACAAGGACGAGACAGTTCC	TCCCGCCTCCTTAAGCATTG
**SOCS1**	NM_001137648.1	CTGCTGGATGCCTGCGGCTT	GGGCCCGGTCGCGGTTTTAA
**TGFB2**	NM_001031045	CAACCCGAGCTCCAAGCGCA	AGCCCCGCGTCTGCACATTC
**STAT1**	NM_001012914.1	ACTGCATGCATTGGTGGCCCA	GCTGACGAACTTGCTGCAGGC
**IBDV VP4**	M97346	GGCTGGTCCCGGAGCATTCG	GGTCCACGTTGGCTGCTGCT
**NAF1**	XM_015285318.1	GAGTGTGAGGTGGAAGTGATT	TTCAGTATCGTGGGATGTTGTAG
**ARFGAP3**	XM_015290471.1	GCATCACCTCAACACAAAGAAG	GCTGGTTCTGGCTCTGATAATA

Supplementary Table 5: Excel spreadsheet workbook file (.XLS) with worksheets showing **Information on Deposited Microarray Data.** Data (.CEL) files as deposited in ArrayExpress for current (E-MTAB-4028) and previous (E-MTAB-3711) studies.

Supplementary Figure S1: Sodium butyrate induces SOCS1 expression in CEFs (but not DF-1) and enhances IBDV VP4 RNA expression in CEFs. For the optimization step **(A)**, a time course study was carried out using three concentrations of sodium butyrate (0.5mM, 1.0mM and 2.0mM) over 18 hours. SOCS1 expression was then quantified by qRT-PCR. Addition of 2.0mM of sodium butyrate (SB) yielded a 2.5-fold increase in SOCS1 expression in CEFs after 18 hours. **(B)** In the presence of sodium butyrate (2 mM) DF-1 were infected with IBDV for 2 h then incubated for 16 h. Incubation of DF-1 with sodium butyrate did not induce any significant changes in SOCS1 transcript levels. Sodium butyrate significantly enhanced the expression levels of VP4 transcripts **(C)**, SOCS1 mRNA **(D)** and ISGs such as IFIT5 **(E)** in IBDV-infected CEFs 18h post infection. All qRT-PCR data are from three independent experiments and are expressed as mean ± SEM. An unpaired t-test with Welch's correction (Two-tailed) was used in A-D (**, P <0.01) and in E a Two-way Anova with Bonferroni's comparison test was used to analyse the data (****, P <0.0001).

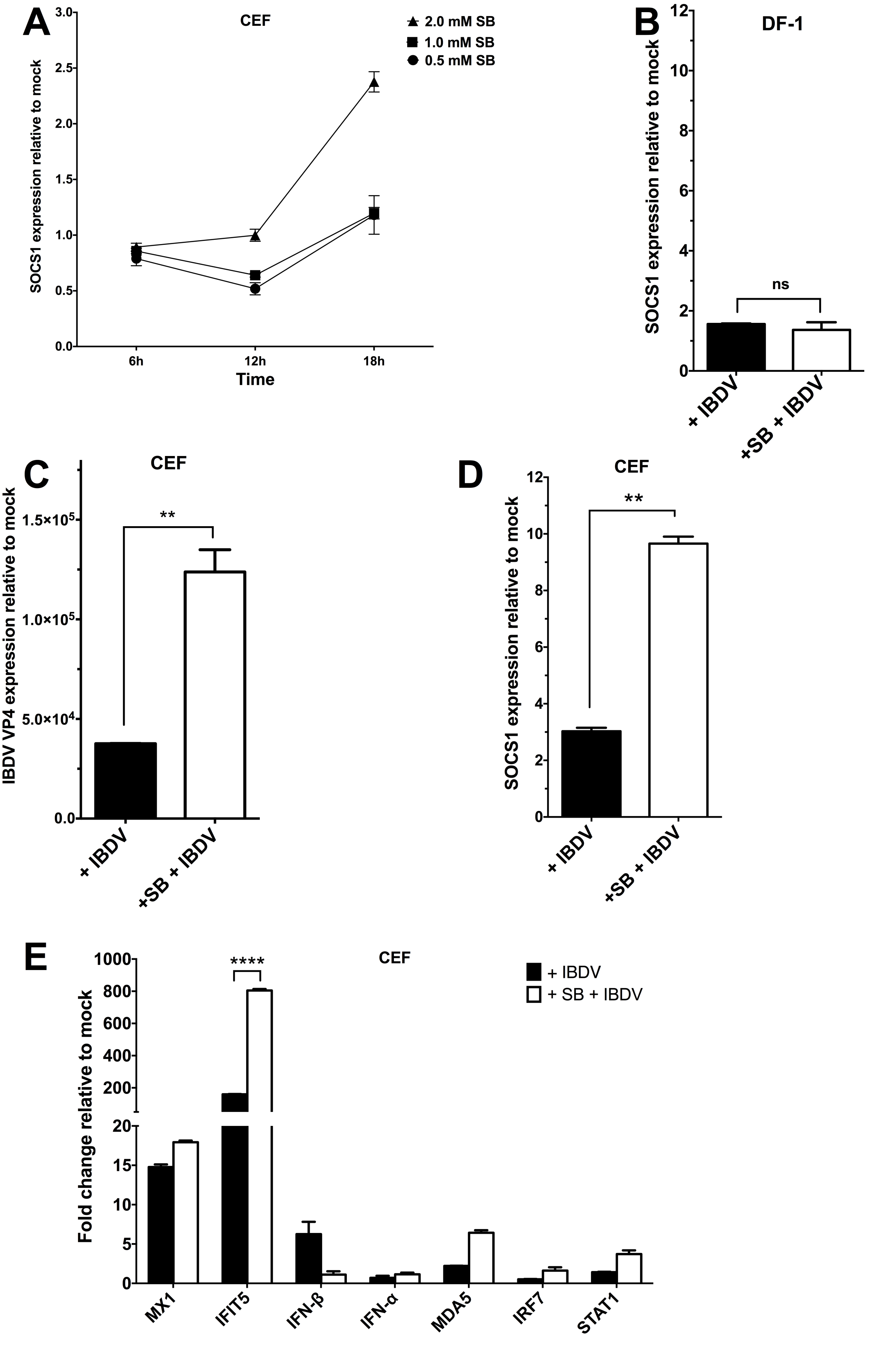
A
CEF
2.0 mM SB
1.0 mM SB
0.5 mM SB
SOCS1 expression relative to mock
3.0
2.5
2.0
1.5
1.0
0.5
0.0
6h
12h
18h
Time
B
DF-1
SOCS1 expression relative to mock
12
10
8
6
4
2
0
ns
+ IBDV
+SB + IBDV
C
CEF
IBDV VP4 expression relative to mock
1.5×10^5
1.0×10^5
5.0×10^4
0.0
**
+ IBDV
+SB + IBDV
D
CEF
SOCS1 expression relative to mock
12
10
8
6
4
2
0
**
+ IBDV
+SB + IBDV
E
CEF
+ IBDV
+ SB + IBDV
Fold change relative to mock
1000
800
600
400
200
20
15
10
5
0
****
MX1
IFIT5
IFN-β
IFN-α
MDA5
IRF7
STAT1

Supplementary Figure S2: Overexpression of SOCS1 inhibits IBDV-induced STAT1 phosphorylation in DF-1 cells. DF-1 cells were either mock-transfected or transfected with a SOCS1 expression vector for 48h and then infected with the IBDV PBG98 strain, at an MOI of 5, for 16h. Total protein lysates were subjected to Western blot analysis using anti-IBDV VP2/3 (Skinner MA, unpublished), anti-STAT1 (BD Biosciences), anti-phospho STAT1 (Millipore), anti-SOCS1 (Sigma) and anti-GAPDH (Ambion) antibodies. Full-length blots for these cropped images are presented in Supplementary Fig. S5 online.

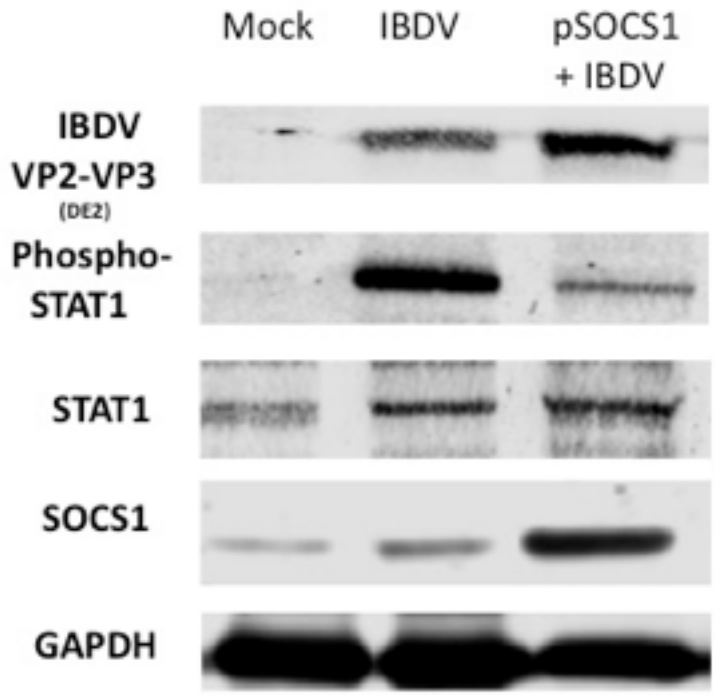

Supplementary Figure S3: BLAST analysis of 19 base siSOCS1 sequence against Gallus gallus nucleotide sequences at NCBI. (A) Top 9 hits (of 171 from default maximum 100 sequences). The top 4 all represent SOCS1; the fifth is from a non-coding genomic scaffold; the next 4 represent NAF1 and ARFGAP3. (B) The SOCS1 hit showing 19/19 base identity. (C) NAF1 and ARFGAP3 hits showing 15/19 base identity.

A

Description	Max score	Total score	Query cover	E value	Ident	Accession
PREDICTED: Gallus gallus suppressor of cytokine signaling 1 (SOCS1), transcript variant X1, mRNA	38.2	38.2	100%	0.006	100%	XM_015294233.1
Gallus gallus suppressor of cytokine signaling 1 (SOCS1), mRNA	38.2	38.2	100%	0.006	100%	NM_001137648.1
Gallus gallus finished cDNA, clone ChEST512p4	38.2	38.2	100%	0.006	100%	BX933215.2
Gallus gallus finished cDNA, clone ChEST419m16	38.2	38.2	100%	0.006	100%	BX932033.1
Gallus gallus BAC clone CH261-57M24 from chromosome z, complete sequence	32.2	105	84%	0.36	100%	AC197851.3
PREDICTED: Gallus gallus nuclear assembly factor 1 ribonucleoprotein (NAF1), transcript variant X2, misc_RNA	30.2	30.2	78%	1.4	100%	XR_001466252.1
PREDICTED: Gallus gallus nuclear assembly factor 1 ribonucleoprotein (NAF1), transcript variant X1, mRNA	30.2	30.2	78%	1.4	100%	XM_015285318.1
PREDICTED: Gallus gallus ADP-ribosylation factor GTPase activating protein 3 (ARFGAP3), transcript variant X2, mRNA	30.2	30.2	78%	1.4	100%	XM_015290471.1
PREDICTED: Gallus gallus ADP-ribosylation factor GTPase activating protein 3 (ARFGAP3), transcript variant X1, mRNA	30.2	30.2	78%	1.4	100%	XM_015290461.1

B

PREDICTED: Gallus gallus suppressor of cytokine signaling 1 (SOCS1), transcript variant X1, mRNA
Sequence ID: **XM_015294233.1** Length: 1077 Number of Matches: 1
Range 1: 473 to 491

Score	Expect	Identities	Gaps	Strand	Frame
38.2 bits(19)	0.006()	19/19(100%)	0/19(0%)	Plus/Plus	

Features:

```
Query  1    CGCAGAAGAATTGTTTCTT  19
            |||||||||||||||||||
Sbjct  473  CGCAGAAGAATTGTTTCTT  491
```

C

PREDICTED: Gallus gallus nuclear assembly factor 1 ribonucleoprotein (NAF1), transcript variant X1, mRNA
Sequence ID: **XM_015285318.1** Length: 10444 Number of Matches: 1
Range 1: 5483 to 5497

Score	Expect	Identities	Gaps	Strand	Frame
30.2 bits(15)	1.4()	15/15(100%)	0/15(0%)	Plus/Plus	

Features:

```
Query  5     GAAGAATTGTTTCTT  19
             |||||||||||||||
Sbjct  5483  GAAGAATTGTTTCTT  5497
```

PREDICTED: Gallus gallus ADP-ribosylation factor GTPase activating protein 3 (ARFGAP3), transcript variant X2, mRNA
Sequence ID: **XM_015290471.1** Length: 2707 Number of Matches: 1
Range 1: 2225 to 2239

Score	Expect	Identities	Gaps	Strand	Frame
30.2 bits(15)	1.4()	15/15(100%)	0/15(0%)	Plus/Minus	

Features:

```
Query  2     GCAGAAGAATTGTTT  16
             |||||||||||||||
Sbjct  2239  GCAGAAGAATTGTTT  2225
```

Supplementary Figure S4: siSOCS1 does not suppress levels of NAF1 & ARFGAP3 RNA. DF-1 cells were mock transfected or transfected with siRNA specific for SOCS1 for 42h and treated with chIFN-α (1000 units/ml) for 6h or infected with the IBDV PBG98 strain for 16h. Extracted total RNA was subjected to reverse transcription followed by quantitative PCR using specific primer sets for SOCS1, NAF1 or ARFGAP3, normalized against GAPDH (using the ΔΔCt method). Data in A-F are representative from three independent experiments.

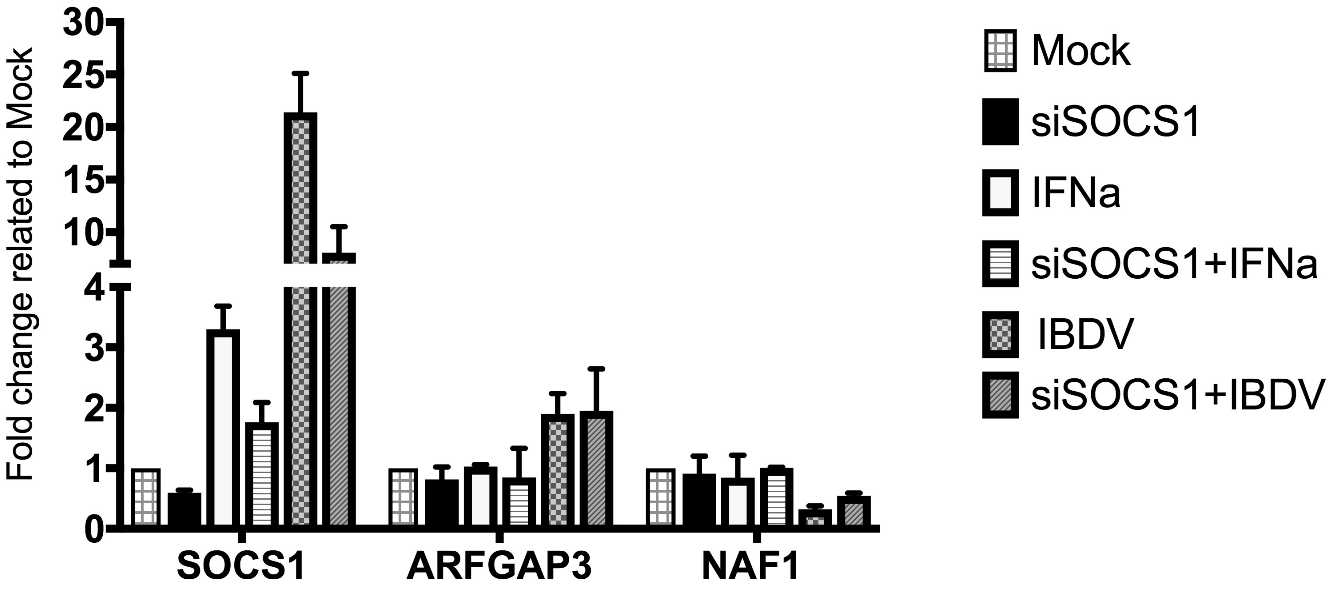

Supplementary Figure S5: Full-size blots. Un-cropped images for Figure 3 (G) & (H), Figure 4 (C) and Supplementary Fig S2. Details are as described in legends for those Figures.

Figure 3G

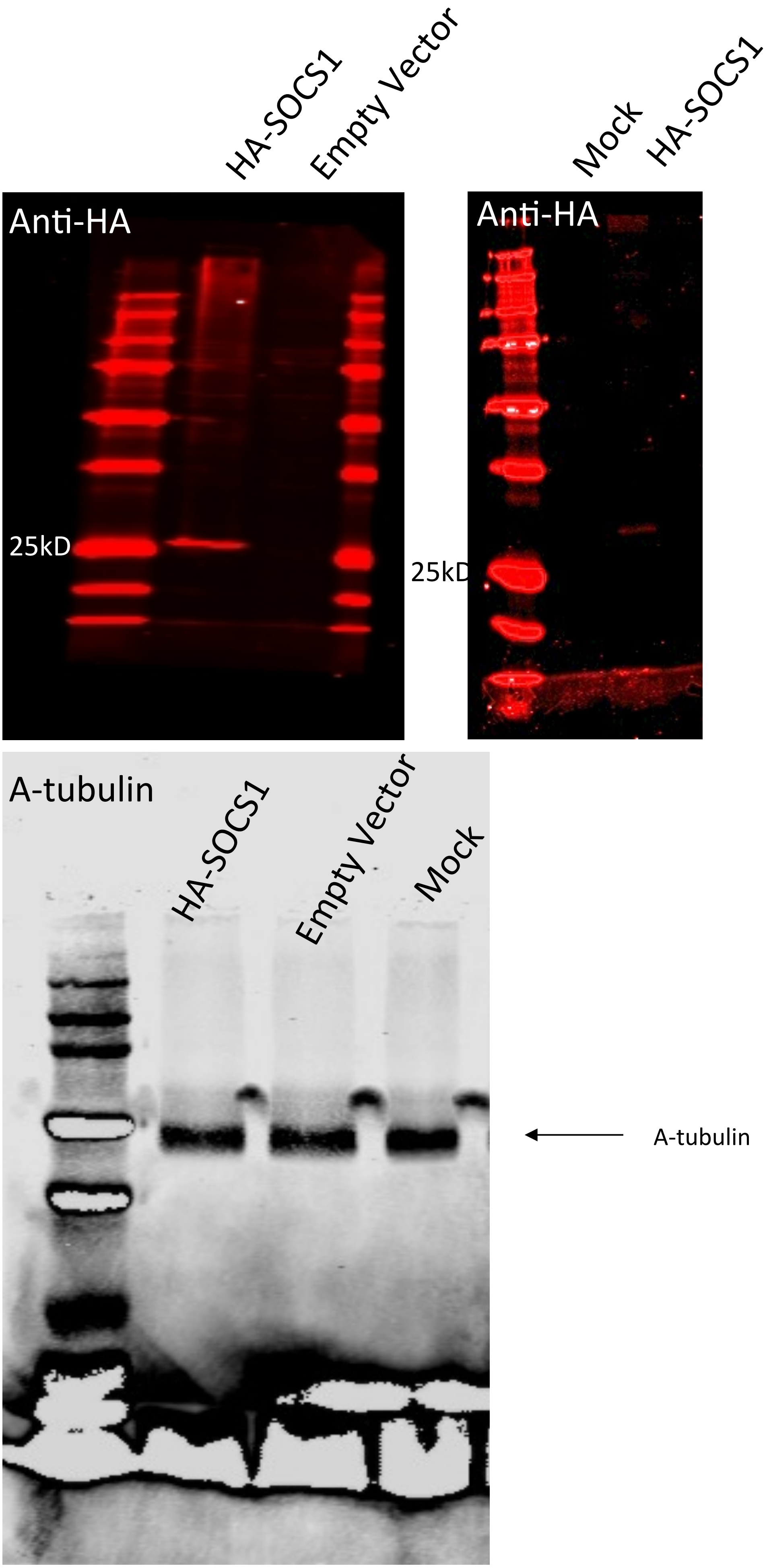

HA-SOCS1
Empty Vector
Mock
HA-SOCS1
Anti-HA
25kD
Anti-HA
25kD
A-tubulin
HA-SOCS1
Empty Vector
Mock
A-tubulin

Figure 3H

cSOCS1 siRNA

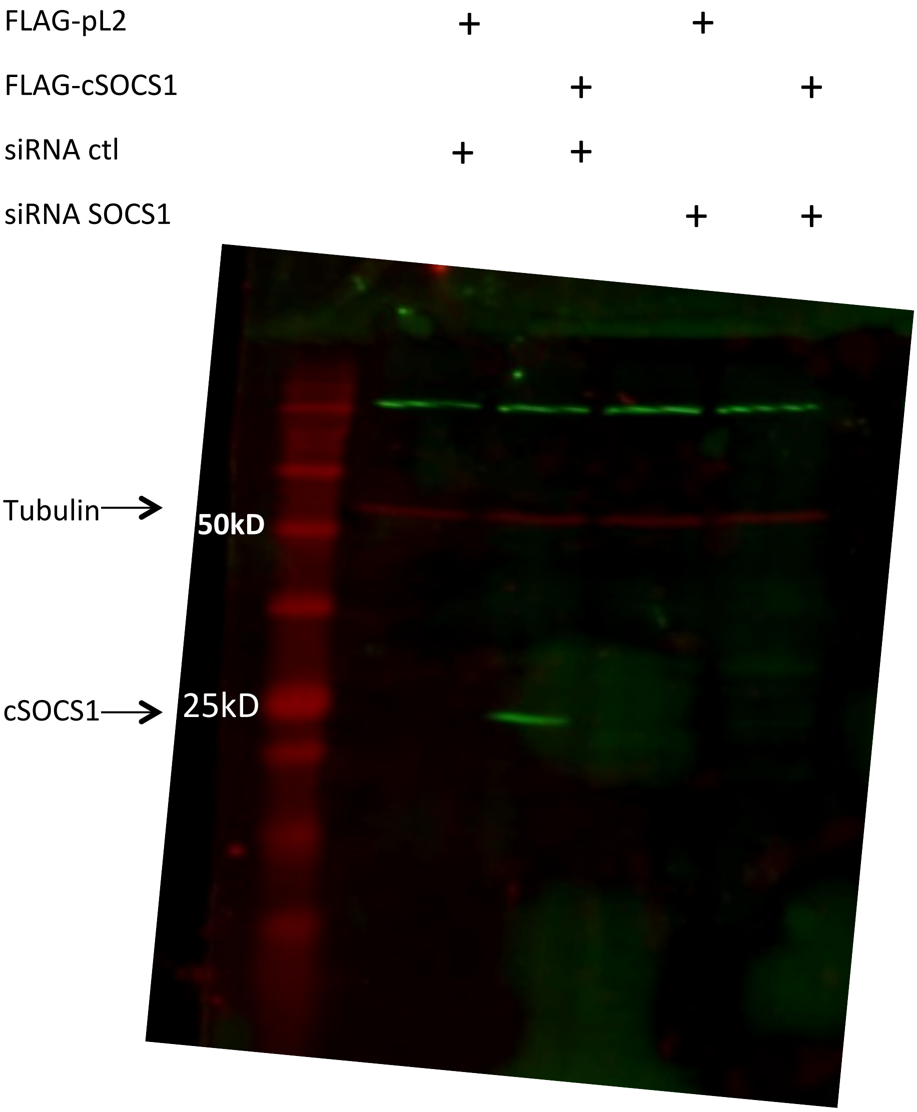

Figure 4C

Mutant cSOCS1 expression

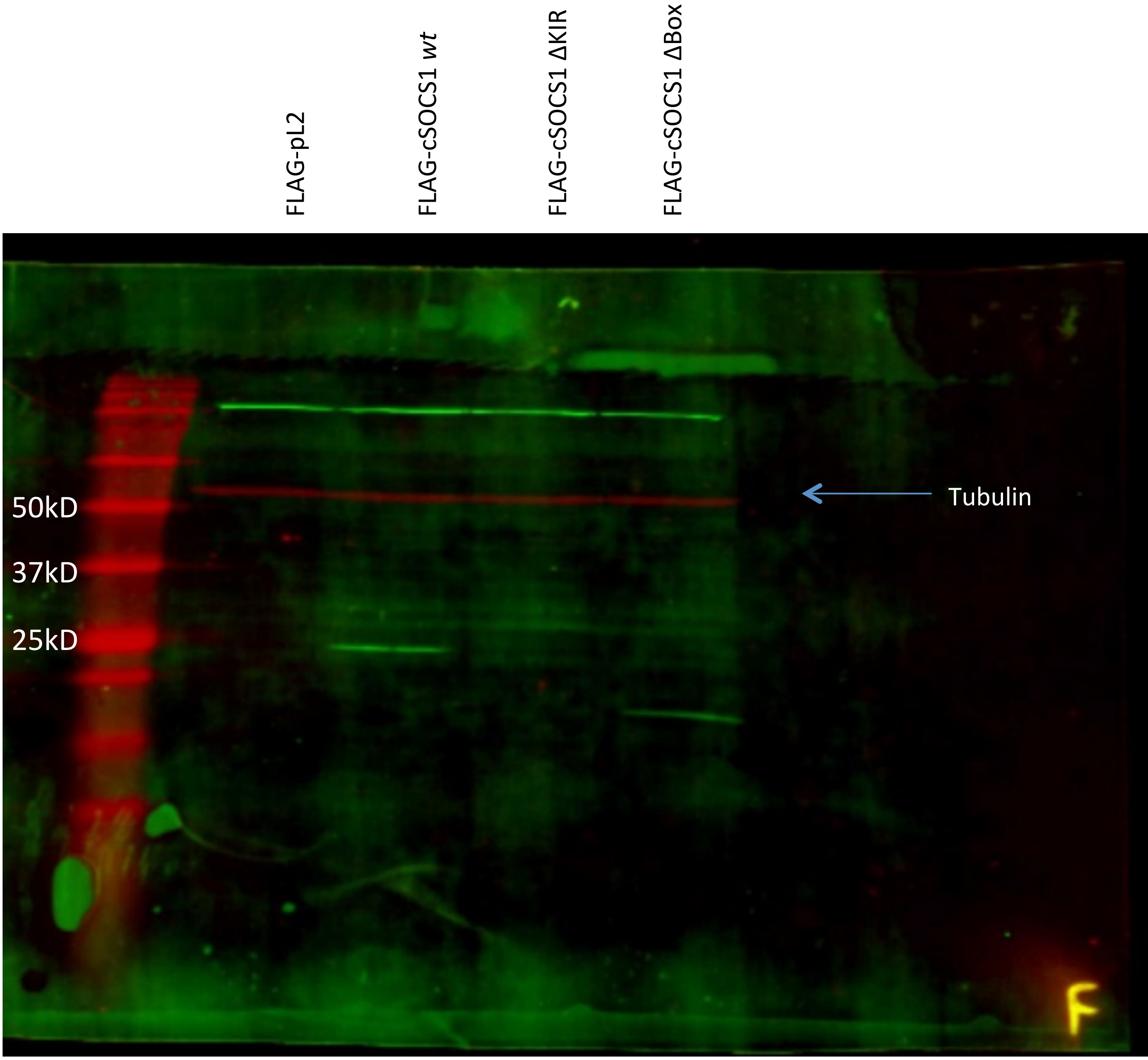

Figure S4

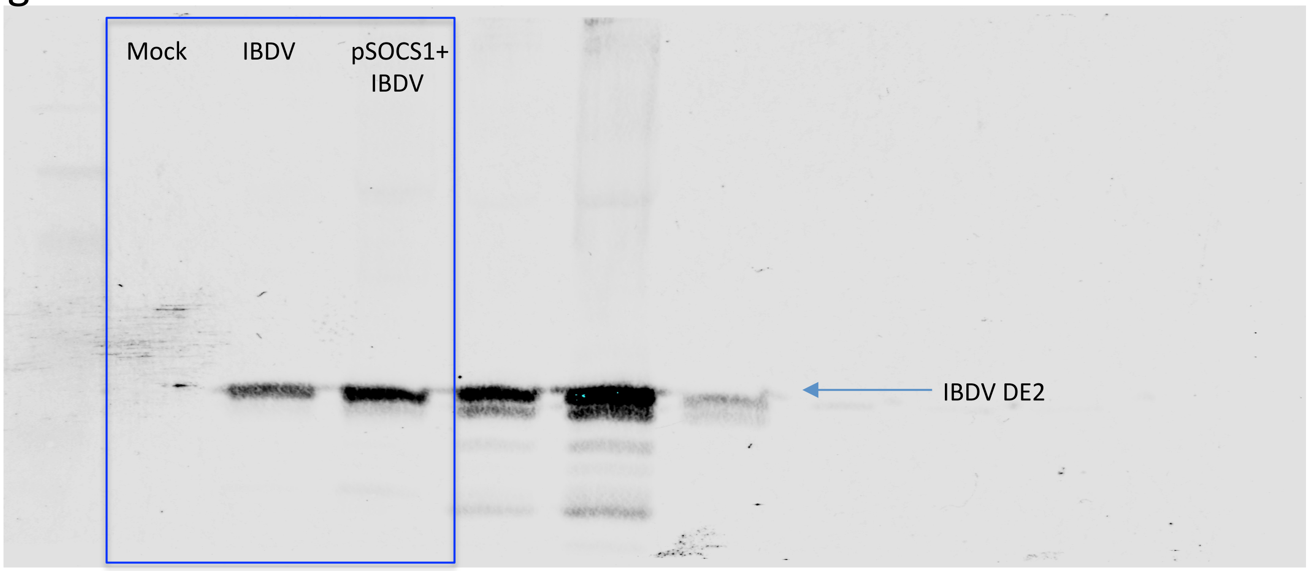
Mock
IBDV
pSOCS1+ IBDV
IBDV DE2

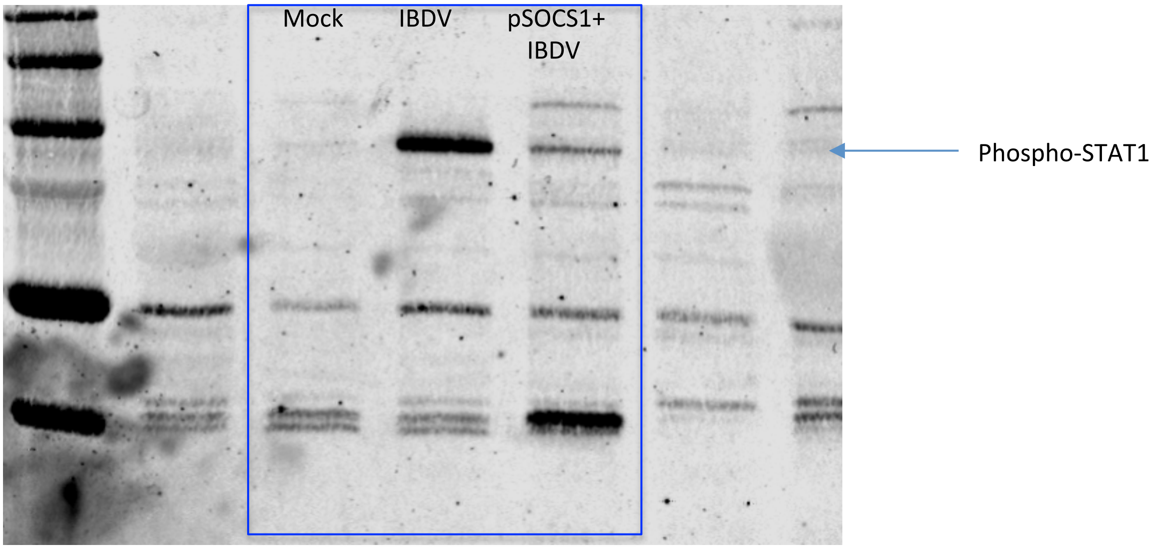
Mock
IBDV
pSOCS1+ IBDV
Phospho-STAT1

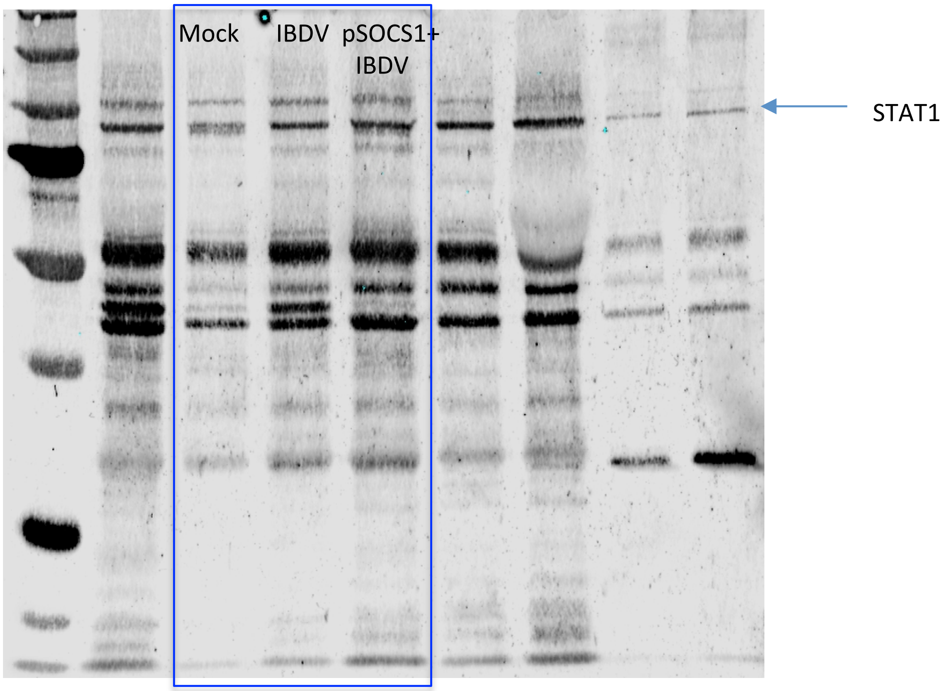
Mock
IBDV
pSOCS1+ IBDV
STAT1

Figure S4

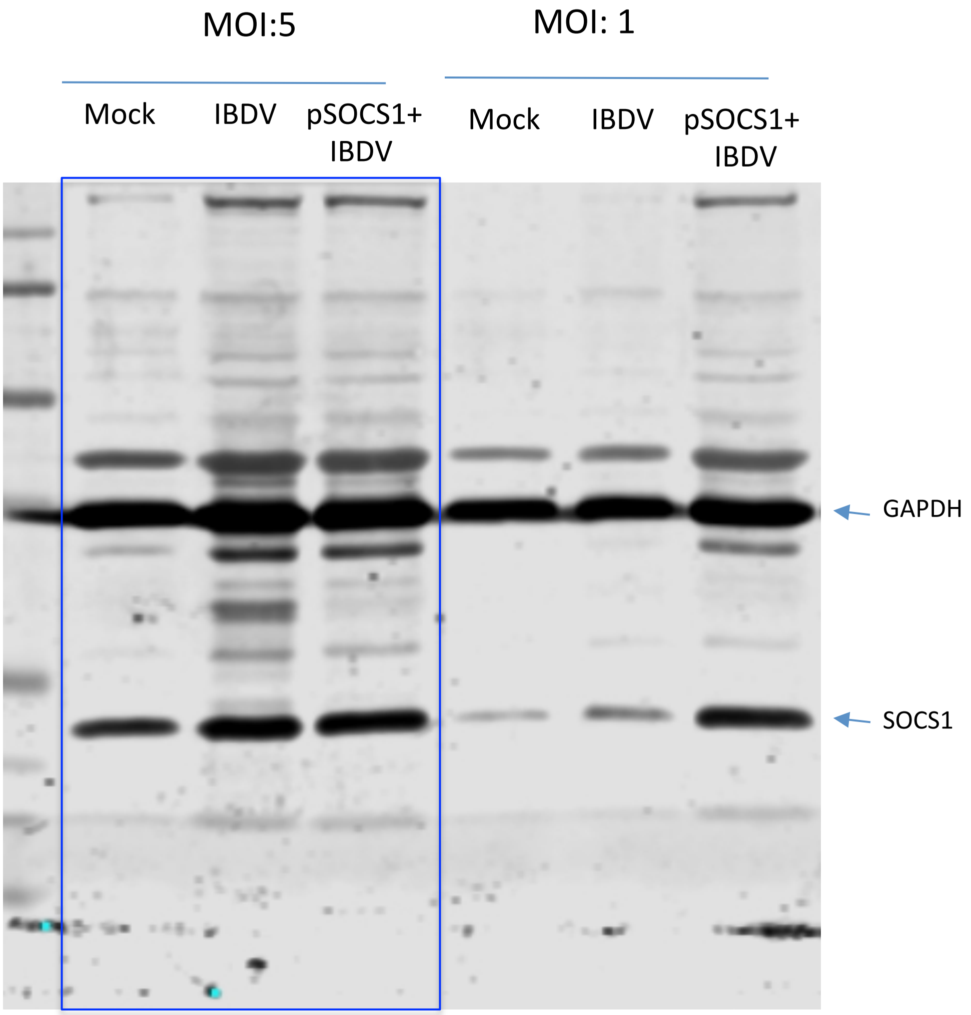

MOI:5
MOI: 1
Mock
IBDV
pSOCS1+ IBDV
Mock
IBDV
pSOCS1+ IBDV
GAPDH
SOCS1
